# The surface-agnostic advantage for peri-implant health: UV photofunctionalization as a positive-sum strategy for biofilm suppression and soft-tissue barrier—a systematic review with qualitative synthesis

**DOI:** 10.1186/s40729-026-00695-1

**Published:** 2026-06-17

**Authors:** Keiji Komatsu, Jasper Kim, Nicholas Her, Ryan Alpers, Natsumi Saito, Rune Shibata, Irina Fedorowicz, Sei Jin Kim, Naryung Kim, Tammy Lu, Andrew Tran, Jisub Lim, Wakako Sakaguchi, Takuma Sato, Shugo Haga, Takanori Matsuura, Wonhee Park, Takahiro Ogawa

**Affiliations:** 1 Weintraub Center for Reconstructive Biotechnology, UCLA School of Dentistry, 10833 Le Conte Avenue B3-087, Box 951668, Los Angeles, CA 90095-1668 USA; 2 Department of Lifetime Oral Health Care Sciences, Graduate School of Medical and Dental Sciences, Institute of Science Tokyo, Tokyo, Japan; 3 Department of Periodontology, Graduate School of Medical and Dental Sciences, Institute of Science Tokyo, Tokyo, Japan; 4 Department of Environmental Pathology, Kanagawa Dental University, Yokosuka, Japan; 5 Department of Orthodontics, Aichi Gakuin University School of Dentistry, Nagoya, Japan; 6 Department of Orthodontics, School of Dentistry, Showa Medical University, Tokyo, Japan; 7 Department of Dentistry, College of Medicine, Hanyang University, Seoul, Korea; 8 Division of Regenerative and Reconstructive Sciences, UCLA School of Dentistry, Los Angeles, CA USA

**Keywords:** Peri-implantitis, Peri-implant mucosal seal, High-energy hydrophilic surface, Hydrocarbon, Bacterial adhesion

## Abstract

**Purpose:**

Long-term dental implant success depends on a biologic “race to the surface,” in which osteogenic cells, peri-implant soft-tissue cells, and bacterial pathogens compete for early dominance at the implant–tissue interface. Because implant surface design is often optimized for one objective at the expense of another (e.g., micro-roughness to accelerate osteoconductivity but with increased plaque-retention risk; relatively smooth transmucosal surfaces to discourage bacterial attachment despite uncertainty regarding optimal soft-tissue integration), strategies that enhance peri-implant health without forcing topographical trade-offs are needed. Ultraviolet (UV) photofunctionalization—by removing storage-acquired hydrocarbons (“biological aging”) and converting surfaces to a high-energy, superhydrophilic state—has been proposed as a chairside, topography-preserving approach to improve interfacial biology. This systematic review evaluates whether UV photofunctionalization of titanium and zirconia surfaces provides clinically relevant advantages for (1) reduction of bacterial attachment and biofilm formation, (2) peri-implant soft-tissue responses relevant to mucosal sealing, and (3) human clinical outcomes.

**Methods:**

After systematic literature search, screening and full-text evaluation, a total of 34 articles, including 9 bacterial/biofilm, 13 soft-tissue (1 overlapping between bacterial and soft-tissue), and 13 clinical studies were selected. Findings were synthesized qualitatively with attention to protocol heterogeneity (UV wavelength band, exposure duration, device configuration, and material and surface types).

**Results:**

Across experimental models, UV photofunctionalization most consistently reduced early bacterial attachment and/or early biofilm accumulation across several titanium surface topographies, supporting an early anti-adhesive and biofilm-suppressive phenotype. Soft-tissue studies generally demonstrated enhanced fibroblast/epithelial attachment, spreading, and functional behaviors relevant to sealing on both titanium and zirconia, although the optimal underlying topography for soft-tissue integration remains unresolved. Clinically, the most consistent signal was accelerated and enhanced implant stability development, while selected studies also suggested favorable trends in peri-implant soft-tissue parameters and/or crestal bone maintenance. However, clinical outcomes remained variable and were limited by heterogeneity in UV protocols, surface systems, endpoints, and follow-up duration.

**Conclusions:**

UV photofunctionalization can be conceptualized as a surface-agnostic physicochemical reactivation technology: a topography-preserving enhancement that restores high surface energy and favorable surface chemistry without altering the underlying surface architecture. Current evidence for this concept is strongest for titanium, whereas supportive evidence for zirconia is emerging primarily from soft-tissue and interface-focused models. This interface-first, positive-sum strategy may allow clinicians to select zone-specific topographies (e.g., smooth transmucosal regions and rough endosteal regions) while maximizing soft-tissue affinity and suppressing early colonization. Although current clinical evidence most strongly supports accelerated osseointegration/stability development, further longitudinal studies with standardized peri-implant health, microbiologic, and mucosal inflammatory endpoints are needed to clarify the long-term translational impact of UV photofunctionalization on peri-implant disease prevention.

**Graphical abstract:**

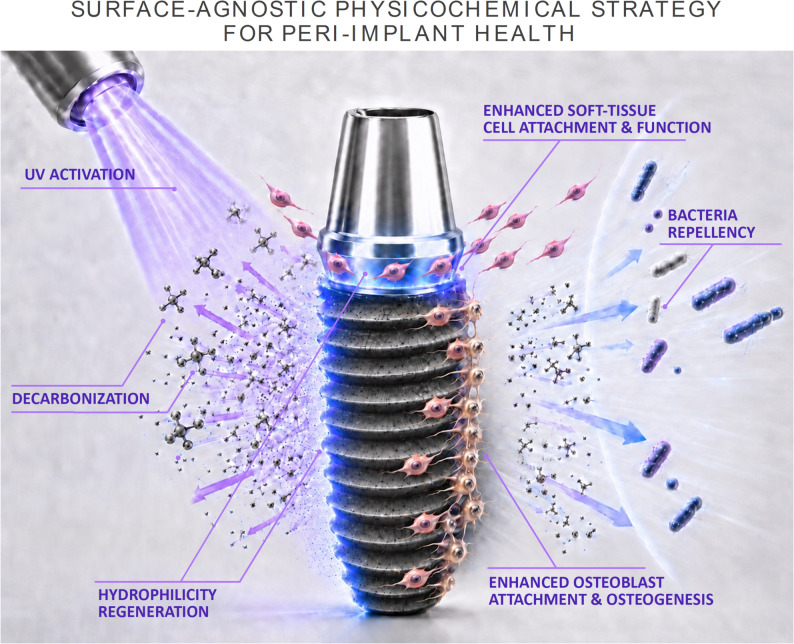

## Introduction

Dental implant therapy demonstrates high long-term success; however, biological complications remain common in routine practice and are strongly linked to plaque biofilm accumulation and peri-implant inflammation. Contemporary classification frameworks describe peri-implantitis as a plaque-associated pathological condition characterized by inflammation of the peri-implant mucosa with progressive supporting bone loss, typically presenting with bleeding on probing and/or suppuration, increased probing depths, and radiographic bone loss [1]. Peri-implant mucositis affects nearly half of patients (46%) and roughly one in five implants (21%) experience peri-implantitis [2]. As a result, strategies that limit early bacterial colonization and promote a stable peri-implant mucosal barrier are increasingly recognized as central—not optional—to long-term implant health.

**Fig. 1:**
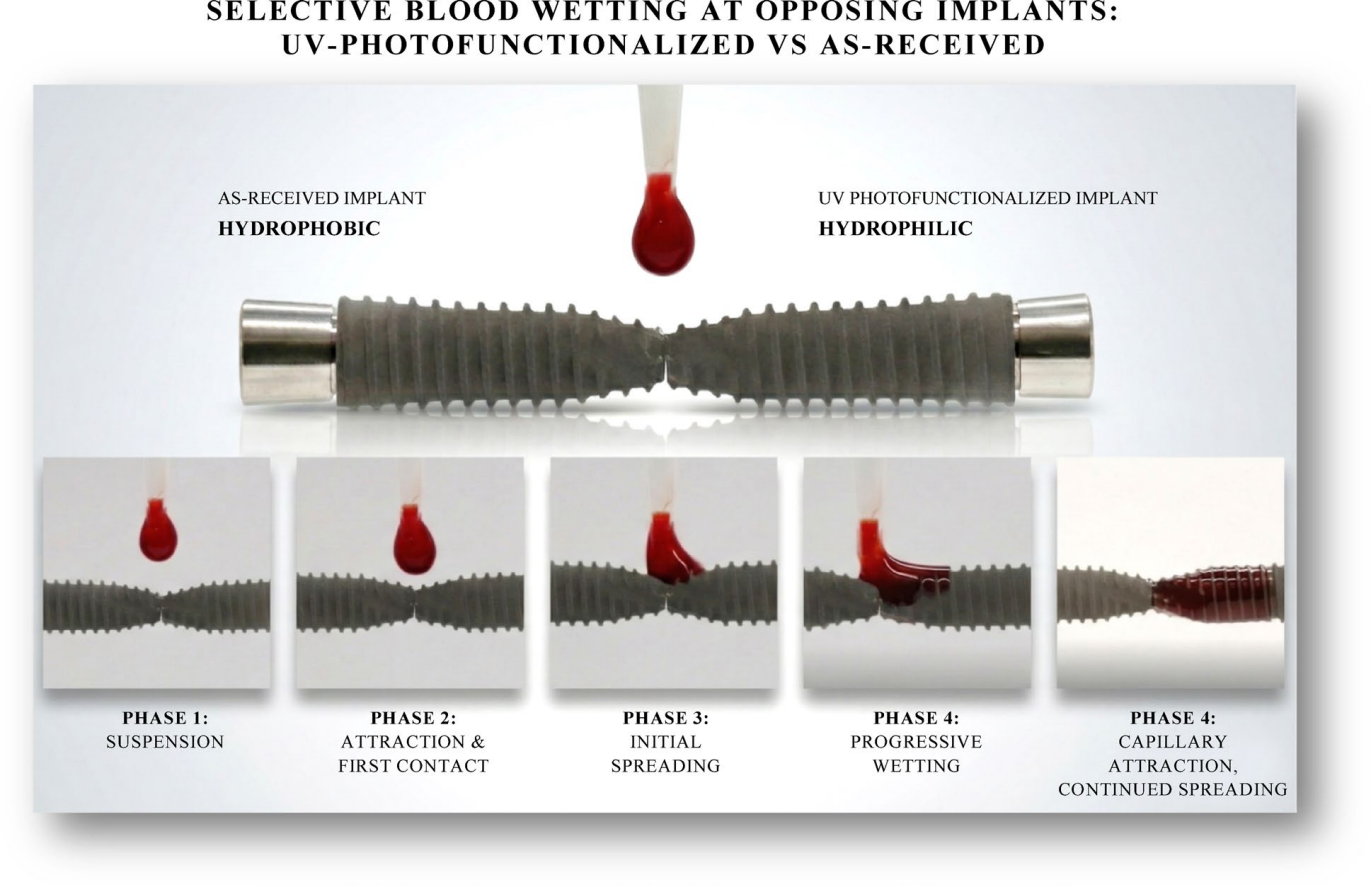
Selective blood wetting at opposing implants: UV-photofunctionalized vs. as-received. Sequential images showing whole-blood behavior between two opposing dental implant tips positioned face-to-face, with one implant photofunctionalized by UV and the other left as-received. The time series captures distinct phases of wetting: Phase 1 (Suspension), blood is held between the tips; Phase 2 (Attraction and first contact), blood is preferentially drawn toward the UV-treated surface before contact; Phase 3 (Initial spreading), blood rapidly spreads upon contacting the UV-treated implant; Phase 4 (Progressive wetting), wetting expands over the UV-treated surface while the as-received surface remains unwetted; and Phase 5 (Saturation and continued spreading), sustained wetting and further spreading continue on the UV-treated implant. The sequence illustrates an “all-or-nothing”/selective wetting phenotype in which UV photofunctionalization dominates the initial blood–surface interaction at the implant interface

**Fig. 2:**
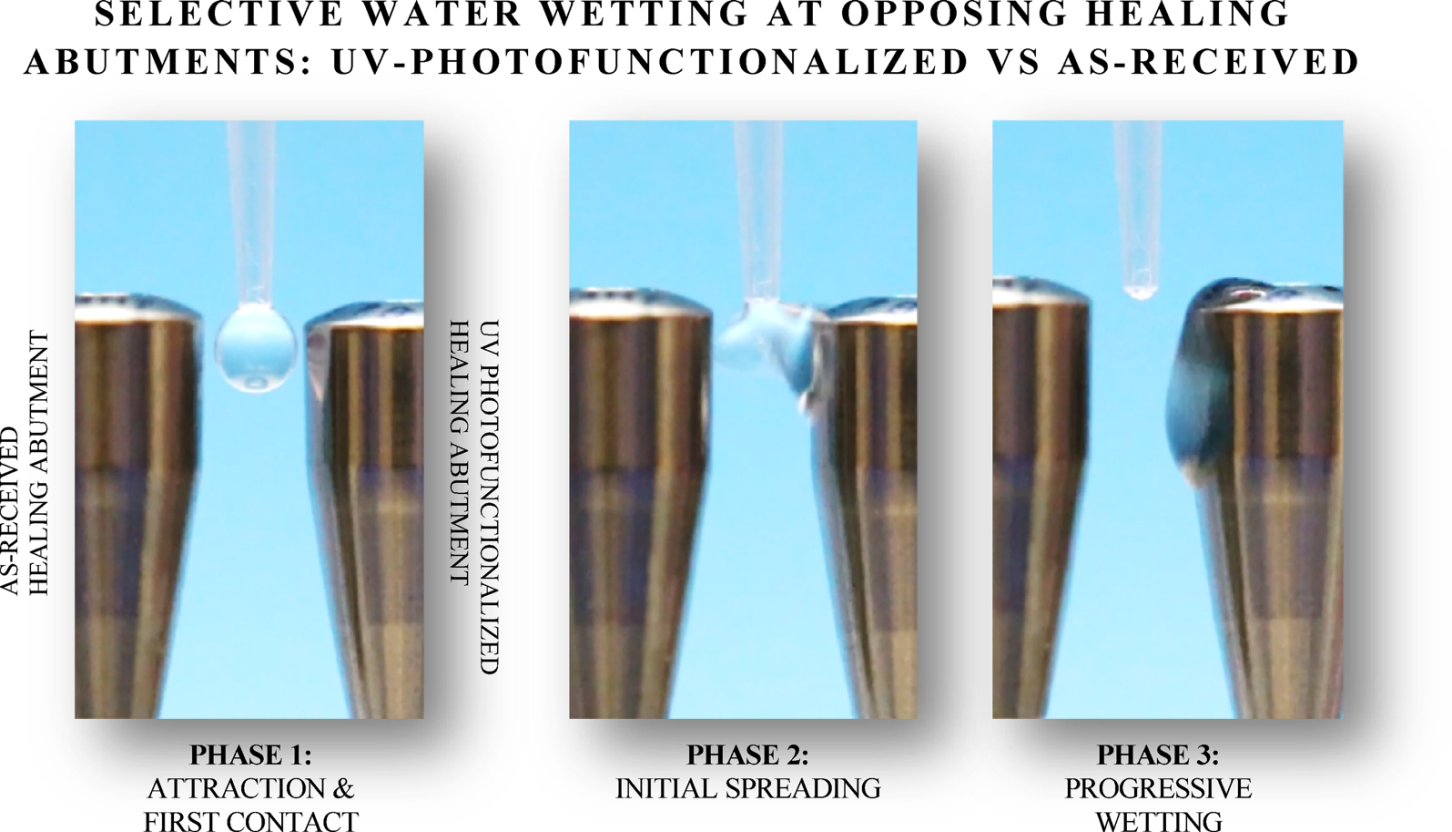
Selective water wetting at opposing healing abutments: UV-photofunctionalized vs. as-received. Sequential images showing water behavior between two opposing healing abutments, with one abutment photofunctionalized by UV and the other left as-received. The time series captures staged wetting dynamics: Phase 1 (Attraction and first contact), the droplet is preferentially drawn toward the UV-treated abutment prior to contact; Phase 2 (Initial spreading), water rapidly spreads upon contacting the UV-treated surface; Phase 3 (Progressive wetting), wetting continues to expand along the UV-treated abutment while the as-received abutment remains unwetted. The sequence illustrates a selective (one-sided) wetting phenotype, consistent with UV-induced restoration of a high-energy, hydrophilic surface state on the treated abutment

Ultraviolet (UV) photofunctionalization has emerged as a practical, chairside-compatible strategy for physicochemical reactivation of titanium surfaces [3–7]. The prevailing concept is that UV treatment reduces storage-acquired hydrocarbon contamination (“biological aging”) and converts titanium surfaces from hydrophobic to highly hydrophilic states (Figs. 1 and 2), thereby improving protein adsorption and subsequent biological interactions at the implant–tissue interface [3, 8–13]. Although the technology has been widely investigated in the context of accelerated osseointegration [14–20], UV-mediated changes in surface physicochemistry are increasingly recognized as potentially relevant to a broader set of peri-implant biologic processes extending beyond bone anchorage alone. Experimental studies have reported reduced bacterial attachment and suppressed early biofilm accumulation on UV-treated surfaces [21–24], while parallel investigations have demonstrated enhanced fibroblast and epithelial responses relevant to establishment of a functional peri-implant soft-tissue barrier [25, 26]. Clinically, emerging human studies have begun examining whether these combined effects translate into improved peri-implant tissue stability and crestal bone maintenance under routine treatment conditions. Accordingly, the significance of UV photofunctionalization may not lie solely in enhancement of osseointegration itself, but rather in its potential to simultaneously influence three interrelated interface domains central to peri-implant health: microbial colonization, mucosal barrier formation, and biologic/mechanical implant stabilization.

Despite this growing body of work, the evidence base remains fragmented. Existing reviews and meta-analyses have predominantly emphasized osseointegration outcomes in in vitro and animal models [5, 10, 27, 28]. To date, these biologic and clinical dimensions have rarely been synthesized within a unified peri-implant framework. Moreover, UV photofunctionalization protocols vary substantially across studies—including wavelength band, exposure duration, device configuration, timing relative to implantation, and implant/abutment surface type—complicating translation and limiting direct comparison across investigations [14, 20, 27, 29–35]. Therefore, rather than reintroducing UV photofunctionalization itself as a novel concept, the purpose of this review is to systematically reorganize the available evidence into an “interface-first” peri-implant health framework centered on three interrelated defensive domains: suppression of bacterial attachment/biofilm formation, support of the peri-implant soft-tissue seal, and enhancement of biologic/mechanical implant stability. Through this structure, the review aims to clarify what is consistently supported by current evidence, what appears context-dependent, and where major translational uncertainties remain.

Accordingly, this systematic review aims to evaluate the effects of UV photofunctionalization of titanium and zirconia implant/abutment surfaces with a primary focus on (1) bacterial attachment and biofilm formation, (2) peri-implant soft-tissue responses relevant to mucosal sealing, and (3) human clinical outcomes. Osseointegration and mechanistic evidence are summarized to provide biologic rationale and to contextualize findings, but the central objective is to assess whether UV photofunctionalization offers clinically relevant advantages for peri-implant health and disease prevention across study designs and protocol variations.

## Biological trade-offs in implant dentistry

Before considering any “solution” technology, it is important to make the current challenges explicit—especially the scientific blind spots and built-in compromises that are often accepted as inevitable in implant surface design. Mapping potential trade-offs would clarify where present approaches leave residual risk or unmet need, and provides the appropriate rationale for evaluating additive surface strategies that aim to improve peri-implant health without sacrificing established benefits.

### Rough surface–bone mass dilemma: interlocking and differentiation versus proliferation and bone volume

**Fig. 3:**
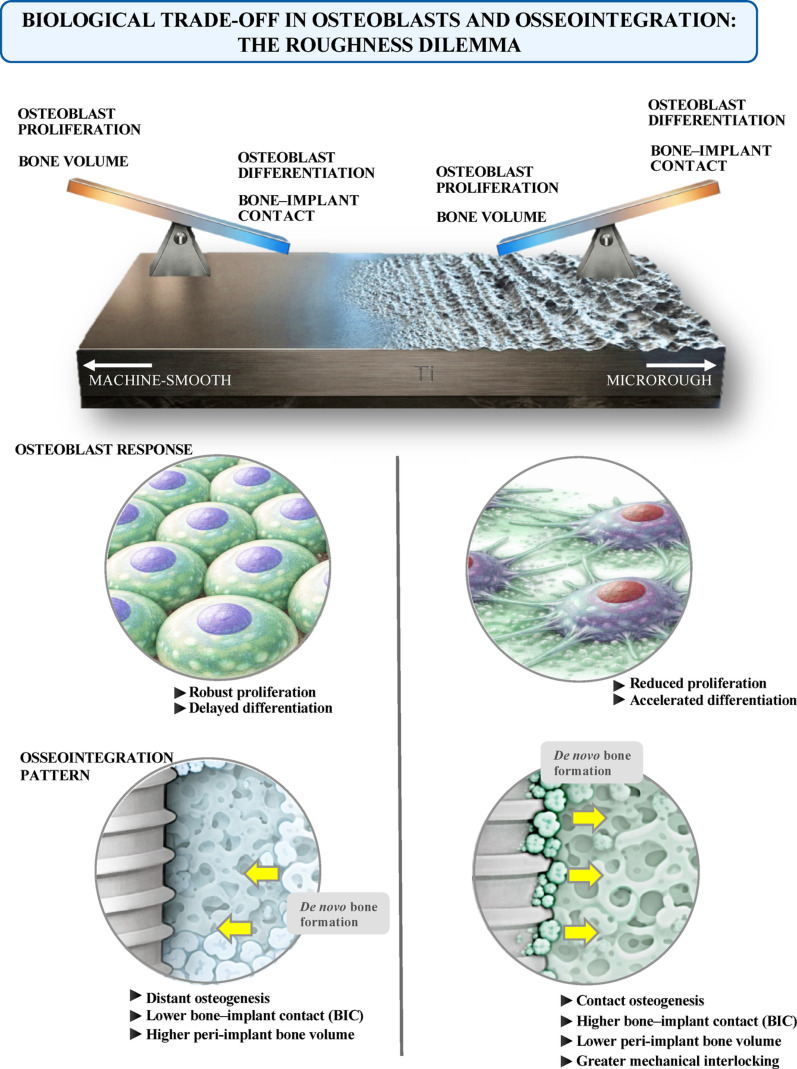
Biological trade-off in osteoblast responses and osseointegration: the roughness dilemma. Conceptual schematic illustrating how implant surface roughness shifts osteoblast behavior and the dominant mode of osseointegration. Smoother surfaces tend to support robust osteoblast proliferation and spreading but can delay maturation/differentiation, often corresponding to a more distant osteogenesis pattern with relatively lower bone–implant contact (BIC) despite greater peri-implant bone volume. In contrast, rougher/microrough surfaces commonly suppress proliferation while promoting accelerated differentiation and matrix maturation, favoring contact osteogenesis with higher BIC and increased mechanical interlocking, but often with a smaller overall peri-implant bone volume. The figure summarizes the inherent design trade-off in conventional surface engineering: roughness can strengthen interfacial anchorage (BIC/interlocking) while potentially reducing the proliferative expansion that contributes to bulk bone volume

Modern implant design has largely been built on the principle that roughened titanium promotes faster and stronger bone anchorage [36–41]. Increased roughness enhances mechanical interlocking at the bone–implant interface and is consistently associated with earlier osteogenic differentiation and maturation of osteoblast-lineage cells [40–49]. Yet this advantage is not biologically “free.” A recurring observation in osteoblast biology is a proliferation–differentiation trade-off: surfaces that drive rapid differentiation may do so at the expense of early proliferation, potentially limiting cellular expansion and surface coverage and, in turn, constraining the volume of newly formed peri-implant bone (Fig. 3) [42, 50–57]. Interestingly, this proliferation restraint is not limited to highly roughened substrates. Osteoblast-lineage cells can exhibit reduced proliferative activity even on relatively smooth titanium, suggesting that titanium itself may shift osteoblast behavior toward earlier maturation rather than expansion, compared with “simple” wound-healing conditions or standard culture on polystyrene [58–60].

This trade-off also aligns with differences in osseointegration pattern and peri-implant bone phenotype. Microrough titanium is commonly associated with contact osteogenesis and the formation of mechanically competent, harder/stiffer interfacial bone [42, 46–49, 61], whereas smoother, machined surfaces more often exhibit distant osteogenesis, in which bone formation occurs at a distance from the implant and the interface can be more readily interposed by soft tissue [62–65]. Thus, many contemporary surfaces implicitly prioritize rapid interfacial bone formation and high bone–implant contact while accepting the possibility of reduced peri-implant bone mass.

Accordingly, implant surface engineering must either identify a roughness “sweet spot” that balances mechanical interlocking, osteoblast maturation, and adequate bone volume, or introduce additive, non-topographical surface dimensions that preserve the osteoconductive advantages of roughness while mitigating its biological penalties.

### Rough surface–bacterial attachment dilemma: osteoconductive architecture versus biofilm permissiveness

A second, clinically consequential trade-off is that surface features optimized for osseointegration can also increase bacterial retention. In the oral environment, microrough topographies may create protected niches that facilitate initial microbial attachment, shield early colonizers from shear forces, and promote the maturation of biofilm communities [23, 66–71]. Once established, biofilms are highly resilient and can elicit sustained inflammation, shifting the peri-implant milieu from regenerative to destructive. Conversely, surfaces engineered primarily to discourage biofilm accumulation often trend toward smoother designs, which may forfeit the osteoconductive “head start” conferred by roughened architecture. Thus, conventional surface engineering frequently forces clinicians and engineers to navigate a compromise between accelerated integration and reduced biofilm permissiveness.

### Abutment and crown-margin dilemma: plaque control versus uncertainty in soft-tissue biology

**Fig. 4:**
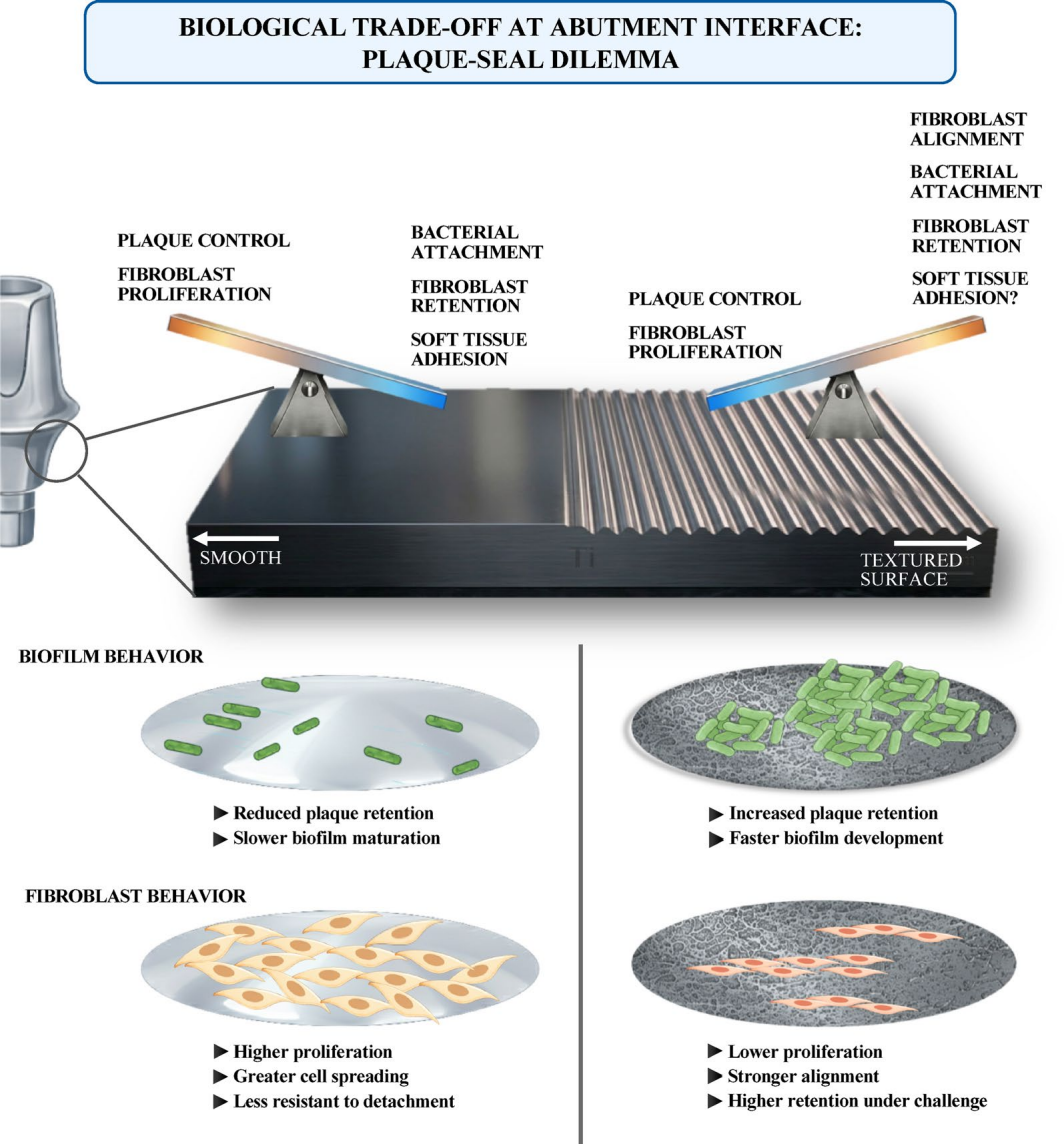
Biological trade-off at the abutment interface: the plaque–seal dilemma. Conceptual schematic illustrating competing priorities in transmucosal/abutment surface design. Smoother, polished surfaces are generally hygiene-favorable because they reduce plaque retention and facilitate cleaning, but they may provide less robust soft-tissue anchorage and a less predictable mucosal seal under functional and inflammatory challenges. In contrast, rougher or textured surfaces can enhance fibroblast alignment and retention and may strengthen soft-tissue adhesion, yet they also increase plaque retention and can accelerate biofilm accumulation. The figure summarizes the fundamental abutment-level trade-off between minimizing biofilm retention (“plaque control”) and maximizing connective-tissue/epithelial attachment (“soft-tissue seal”), which motivates interface-first strategies intended to improve both outcomes without relying on roughness alone

The transmucosal region introduces an additional—and distinct—set of constraints. Abutments and crown margins are commonly finished to relatively smooth surfaces to minimize plaque accumulation and facilitate hygiene, particularly because this zone is continuously exposed to the oral microbiome. Yet smoothing is largely a passive strategy: it reduces mechanical plaque retention but does not actively optimize the biological interactions that govern soft-tissue sealing, including fibroblast attachment strength, epithelial adherence, and resistance to detachment or apical downgrowth (Fig. 4) [72–76]. Moreover, the field has not reached consensus on whether “smoother is always better” for soft-tissue integration, or whether specific micro-/nano-scale features might better support organized connective tissue attachment/adhesion and barrier function [56, 68, 77–83].

This uncertainty highlights a practical limitation in implant prosthodontics. Clinicians can reduce plaque-retentive features at the abutment–implant complex by selecting smoother surfaces, but they have limited tools to actively increase soft-tissue affinity while simultaneously making the interface less permissive to early colonization. Consequently, peri-implant health is often managed through compromise—designing for hygiene-friendliness while accepting incomplete control over the quality and durability of the mucosal seal.

## Overview of UV photofunctionalization: historical context, motivation, and biological rationale

These unresolved trade-offs motivate interest in surface technologies that can enhance early host–surface interactions while discouraging initial microbial colonization, without altering the surface topography selected for a given clinical zone. UV photofunctionalization emerged as one such candidate: a chairside physicochemical treatment that restores surface energy and chemistry, thereby enabling a topography-neutral, positive-sum bio-interface on both smooth transmucosal components and roughened implant fixtures.

### From “bioinert titanium” to “time-dependent loss of bioactivity”

Titanium and titanium alloys have long served as the foundational materials for endosseous implants owing to their favorable mechanical properties and the spontaneous formation of a stable surface oxide layer (primarily TiO_2_), which confers chemical stability and biocompatibility. However, the traditional assumption that titanium surfaces remain intrinsically stable after manufacturing has been revised by studies demonstrating a time-dependent decline in biological performance following fabrication and storage [3, 10, 13, 62, 84–87]. This phenomenon—often termed the biological aging of titanium—describes the progressive loss of a surface’s capacity to attract and retain proteins and osteogenic cells, even in the absence of intentional changes in macroscopic appearance or surface topography [12, 88].

A key mechanistic observation underpinning biological aging is the progressive accumulation of adventitious hydrocarbons on the titanium oxide surface during storage (often detected as increased surface carbon by X-ray photoelectron spectroscopy (XPS)). In parallel, the surface shifts toward hydrophobicity and exhibits altered electrostatic behavior. Importantly, long-term investigations suggest that the decline in biological performance can continue for months and may not be explained solely by wettability changes [85], indicating that multiple physicochemical variables contribute to the “aged” phenotype [62, 82, 89]. These concepts reframed a practical clinical problem: implants are often manufactured, packaged, shipped, and stored for substantial periods before clinical use. If storage-driven surface changes reduce early biological performance, then a clinically deployable method to restore surface bioactivity immediately before use could be valuable.

### Photofunctionalization emerges: UV as a “surface reactivation” strategy

UV photofunctionalization was introduced as a method to reverse storage-related degradation and restore titanium bioactivity [90–92]. The foundational approach exposes titanium to UV irradiation (commonly UVA and/or UVC; more recently vacuum UV (VUV) in specialized systems), producing two clinically meaningful surface-level outcomes [7, 14, 17, 18, 33, 93]:

1. Decarbonization/surface cleaning: UV treatment of titanium can reduce hydrocarbon contamination on titanium oxide, lowering surface carbon content and revealing a cleaner TiO_2_ surface.
2. Wettability conversion: UV treatment can convert titanium from hydrophobic to superhydrophilic, often observed as near-zero contact angles and enhanced spreading of water and blood-like fluids (see Figs. 1 and 2).

These effects can be seen across multiple titanium surface topographies (machined, acid-etched, sandblasted, and nano-surfaces) [16, 51, 55, 88, 94, 95]. The original clinical motivation was therefore straightforward: restore the “fresh” bioactive state of titanium at the chairside, enabling more favorable early biological events.

### Physicochemical basis: what UV changes on titanium surfaces

**(1) Titanium dioxide photocatalysis, UV-mediated photolysis, surface chemistry, and hydrocarbon removal**

The native titanium oxide layer (TiO_2_) is not inert under UV irradiation. In photocatalysis research, UV excitation of TiO_2_ generates reactive species capable of oxidizing organic contaminants, a photochemical basis for the well-known self-cleaning and UV-induced hydrophilicity effects in materials science [66, 96–101]. In implant-relevant contexts, UV therefore functions as a non-contact surface-cleaning approach that decomposes adventitious hydrocarbons. UV can also decompose hydrocarbons via direct photolysis, and recent evidence suggests that this pathway may contribute substantially—potentially exceeding photocatalytic contributions under certain conditions [29, 102]. Across most studies, the atomic percentage of surface carbon decreases markedly after UV treatment, often from 40 to 70% to < 15–20% [30, 31, 102–106].

**(2) Superhydrophilicity: more than “just wetting”**

Photoinduced superhydrophilicity of TiO_2_ has been studied mechanistically in surface science. While details depend on experimental conditions, multiple lines of evidence support that UV exposure can alter the TiO_2_ surface state (e.g., formation of hydroxyl groups, changes in defect states such as oxygen vacancies) and/or remove hydrophobic hydrocarbons, resulting in dramatically increased wettability [31]. From an implant biology standpoint, superhydrophilicity is not merely cosmetic. It can influence the earliest interfacial events—water structuring, protein adsorption kinetics, blood wetting and flow dynamics—that occur before cells ever contact the surface [18, 95, 107–111]. Indeed, the effect can be striking: both blood and water exhibit selective, binary wetting/flow behavior toward UV-photofunctionalized surfaces when paired against untreated as-received implants or abutments (Figs. 1 and 2).

**(3) Electrostatic status and protein adsorption: “surface charge as a bioactivity regulator”**

A major conceptual advance in the photofunctionalization literature is that UV treatment can shift titanium toward a more electropositive surface state, while aged titanium tends to be more electronegative. This electrostatic difference can govern protein adsorption in ways that are not fully predicted by wettability alone.

Mechanistically, electrostatic effects can allow negatively charged proteins and cell membranes to interact more favorably with UV-treated surfaces, potentially reducing reliance on “bridging ions” (e.g., Ca^2+^) for initial adsorption and attachment [84, 108, 109]. This framework is clinically attractive because it suggests UV treatment is not simply “cleaning,” but can modulate interfacial biology by changing the physicochemical rules that govern the earliest recruitment phase [19].

### Effects on osteogenic cells and osseointegration: foundational evidence

**(1) Osteogenic cell responses (in vitro)**

Across multiple studies, UV treatment has been associated with:

- Greater early attachment and spreading of osteoblasts and mesenchymal stem cells on titanium surfaces [7, 14, 15, 94, 112].
- Improved retention/adhesion strength under detachment challenges, consistent with stronger early adhesion complex formation [7, 14, 15, 94].
- Enhanced functional cascades downstream of initial attachment (often discussed as proliferation- and differentiation-related signaling and phenotype expression) [7, 14].

**(2) Osseointegration and mechanical fixation**

Preclinical evidence has linked photofunctionalization to improvements in osteoconductive outcomes, including faster and higher bone–implant contact and improved fixation strength, across different titanium substrates, alloys, and surface textures [3, 4, 11, 14, 113–115]. In biologically and mechanically demanding models such as systemic disease, compromised host conditions, and short-implant or immediate-loading protocols, enhanced interfacial bone formation is frequently accompanied by higher removal torque or push-in/push-out values [20, 32, 34, 106, 113, 116, 117].

### Technology evolution: from conventional UVA/UVC protocols to accelerated VUV approaches

**Fig. 5:**
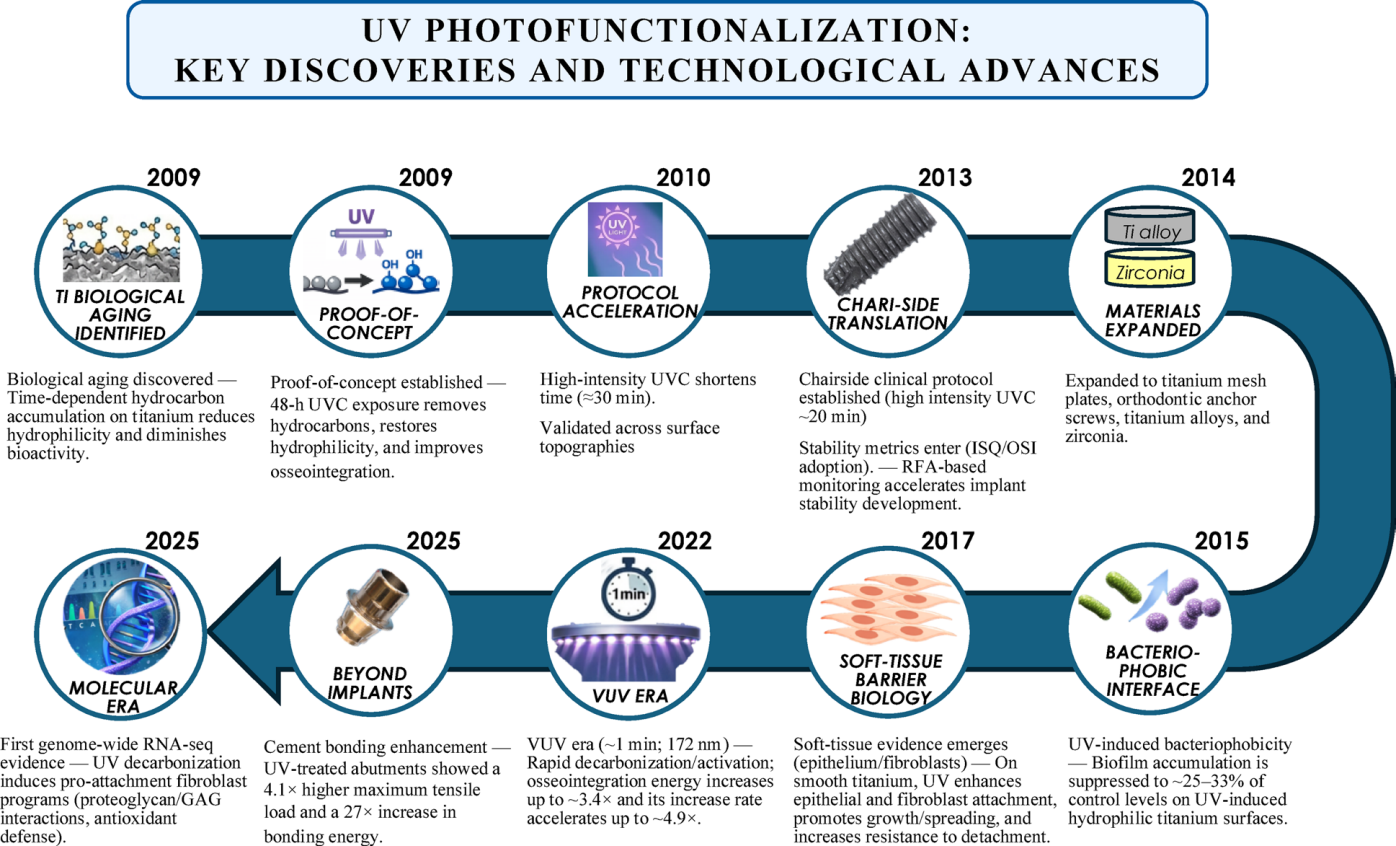
From biological aging to the VUV era: milestones in UV photofunctionalization. Timeline summarizing key discoveries and technological advances in UV photofunctionalization of implant materials and devices. Early work identified “biological aging” of titanium driven by time-dependent hydrocarbon accumulation and loss of hydrophilicity, followed by proof-of-concept UVA/UVC protocols (initially up to 48 h) demonstrating decarbonization, surface reactivation, and improved bone–implant integration. Subsequent innovations accelerated treatment to ~ 30 min using high-intensity UVC and established clinically feasible chairside protocols (~ 20 min) with dental implant translation. The technology then expanded across surface topographies and materials (including titanium alloys and zirconia) and to related devices (e.g., titanium mesh/plates and orthodontic anchor screws). Mechanistic and biological scope broadened with reports of UV-induced bacteriophobicity (suppression of early biofilm accumulation) and emerging soft-tissue evidence showing enhanced epithelial and fibroblast attachment/retention on smooth titanium and zirconia. The timeline culminates in the VUV era (172 nm), enabling ~ 1 min rapid decarbonization with marked increases in osseointegration strength and rate, alongside new applications beyond implants (e.g., enhanced cement bonding) and genome-wide RNA-seq evidence revealing pro-attachment, homeostatic fibroblast programs supporting soft-tissue sealing

A practical limitation of early photofunctionalization protocols was throughput: depending on the UV source, geometry, and intended endpoint, irradiation often required tens of minutes to many hours, which constrained routine chairside use and scaled manufacturing workflows [7, 11, 14, 16]. This “time barrier” became a major driver of technology evolution (Fig. 5). As the field matured, successive generations of devices and protocols focused on preserving the core biological rationale while reducing treatment time and improving clinical feasibility [82].

Milestones included (i) recognition that titanium undergoes time-dependent loss of bioactivity after fabrication/storage (biological aging), (ii) proof that UV treatment can restore bioactivity across materials and surface types, (iii) validation of shorter, chairside-compatible protocols for dental implants [90–92], and (iv) expansion beyond bone-facing applications toward bacteriophobicity, soft-tissue integration, and non-biological interface performance of implant components [118, 119]. Most recently, vacuum ultraviolet (VUV; e.g., 172 nm) systems have been introduced to accelerate organic decomposition and achieve rapid decarbonization—often in one minute—thereby addressing workflow constraints that previously limited translational adoption [29, 30]. In parallel, mechanistic depth has advanced from phenotypic assays to genome-wide molecular readouts (e.g., RNA sequencing), supporting a more comprehensive, pathway-level understanding of UV-enabled bioactivity.

## Systematic review

### Materials and methods

#### Study design

This systematic review was conducted in accordance with the Preferred Reporting Items for Systematic Reviews and Meta-Analyses (PRISMA) guidelines. The objective was to evaluate the effects of UV photofunctionalization on titanium and zirconia dental implant/abutment surfaces across three outcome domains: bacterial/biofilm, soft tissue, and clinical. Because of substantial heterogeneity in experimental models, UV protocols, surface systems, and outcome measures, the review was designed as a structured systematic review with qualitative synthesis rather than a quantitative meta-analysis.

#### Search strategy

A structured electronic search was performed using PubMed (National Library of Medicine). PubMed was selected as the primary database because the field of implant photofunctionalization is highly concentrated within biomedical and dental implant literature indexed in MEDLINE, and the objective of the review was qualitative synthesis and conceptual integration rather than exhaustive bibliometric capture. The literature search was conducted in January 2026 and included all eligible records indexed through that date. The search strategy was developed using a PICO framework and combined Medical Subject Headings (MeSH) with title/abstract keyword searches ([tiab]) to maximize sensitivity and specificity.

- Population (P): Dental implants composed of titanium, titanium alloys, or zirconia.
- Intervention (I): UV photofunctionalization (UVA, UVC, and/or VUV irradiation).
- Comparison (C): Untreated or non-photofunctionalized implant surfaces.
- Outcomes (O): (i) bacterial adhesion/biofilm, (ii) soft-tissue integration (fibroblast/epithelial responses), and (iii) clinical peri-implant outcomes.

To increase outcome specificity, three separate searches were conducted using identical population and intervention blocks and outcome-specific keyword blocks targeting bacterial/biofilm outcomes (*n* = 17 retrieved), soft-tissue outcomes (*n* = 12 retrieved), and clinical outcomes (*n* = 37 retrieved). The complete search strings are provided below to ensure reproducibility.

Bacterial outcomes (*n* = 17 retrieved)

(“photofunctionalization” [tiab] OR “photo-functionalization”[tiab] OR “UV treatment”[tiab] OR “ultraviolet treatment”[tiab] OR “UV light”[tiab] OR “ultraviolet light”[tiab] OR “phototreatment”[tiab]) AND ((“Dental Implants” OR “Dental Implantation, Endosseous” OR “Dental Prosthesis, Implant-Supported” OR “dental implant*”[tiab] OR “oral implant*”[tiab]) AND (“Titanium” OR titanium[tiab] OR “Zirconium” OR zirconia[tiab] OR “zirconium dioxide”[tiab])) AND (“Bacterial Adhesion” OR “Biofilms” OR “Peri-Implantitis” OR bacteri*[tiab] OR biofilm*[tiab] OR “bacterial adhesion”[tiab] OR “bacterial colonization”[tiab] OR antibacterial[tiab] OR periimplantitis[tiab])

Soft-tissue outcomes (*n* = 12 retrieved)

(“photofunctionalization”[tiab] OR “photo-functionalization” [tiab] OR “UV treatment”[tiab] OR “ultraviolet treatment”[tiab] OR “UV light”[tiab] OR “ultraviolet light”[tiab] OR “phototreatment”[tiab]) AND ((“Dental Implants” OR “Dental Implantation, Endosseous” OR “Dental Prosthesis, Implant-Supported” OR “dental implant*”[tiab] OR “oral implant*”[tiab]) AND (“Titanium” OR titanium[tiab] OR “Zirconium” OR zirconia[tiab] OR “zirconium dioxide”[tiab])) AND (“soft tissue integration”[tiab] OR “soft tissue adhesion”[tiab] OR “peri-implant soft tissue”[tiab] OR “keratinized mucosa”[tiab] OR “Gingiva” OR “Periodontium” OR gingiva*[tiab])

Clinical outcomes (*n* = 37 retrieved)

(“photofunctionalization”[tiab] OR “photo-functionalization” [tiab] OR “UV treatment”[tiab] OR “ultraviolet treatment”[tiab] OR “UV light”[tiab] OR “ultraviolet light”[tiab] OR “phototreatment”[tiab]) AND ((“Dental Implants” OR “Dental Implantation, Endosseous” OR “Dental Prosthesis, Implant-Supported” OR “dental implant*”[tiab] OR “oral implant*”[tiab]) AND (“Titanium” OR titanium[tiab] OR “Zirconium” OR zirconia[tiab] OR “zirconium dioxide”[tiab])) AND (human[tiab] OR patient*[tiab] OR “clinical study”[tiab] OR “treatment outcome”[tiab])

####  Additional article identification (citation tracking)

To reduce the risk of missing eligible literature, reference lists of included articles were screened (backward citation tracking). When relevant, studies citing key articles were also reviewed (forward citation tracking). Articles identified through manual screening were included if they met eligibility criteria and evaluated UV photofunctionalization of dental implant/abutment surfaces within the targeted domains. Duplicate records identified through citation tracking were manually removed prior to screening.

**Eligibility criteria**

Inclusion criteria:

- Primary research studies evaluating UV photofunctionalization of titanium or zirconia dental implant/abutment surfaces.
- In vitro, animal, or human clinical studies.
- Studies reporting outcomes in bacterial adhesion/biofilm formation, soft-tissue integration, osseointegration/stability, and/or clinical peri-implant outcomes.
- Peer-reviewed publications in English.

Exclusion criteria:

- Non-implant studies.
- Review articles, single-patient case reports, editorials, or other non-primary research.
- Case series were eligible for inclusion when they reported predefined outcomes relevant to the bacterial/biofilm, soft-tissue, or clinical domains.
- Studies not evaluating UV-mediated surface treatment.
- Studies without outcomes relevant to the predefined domains.

####  Study selection

**Fig. 6:**
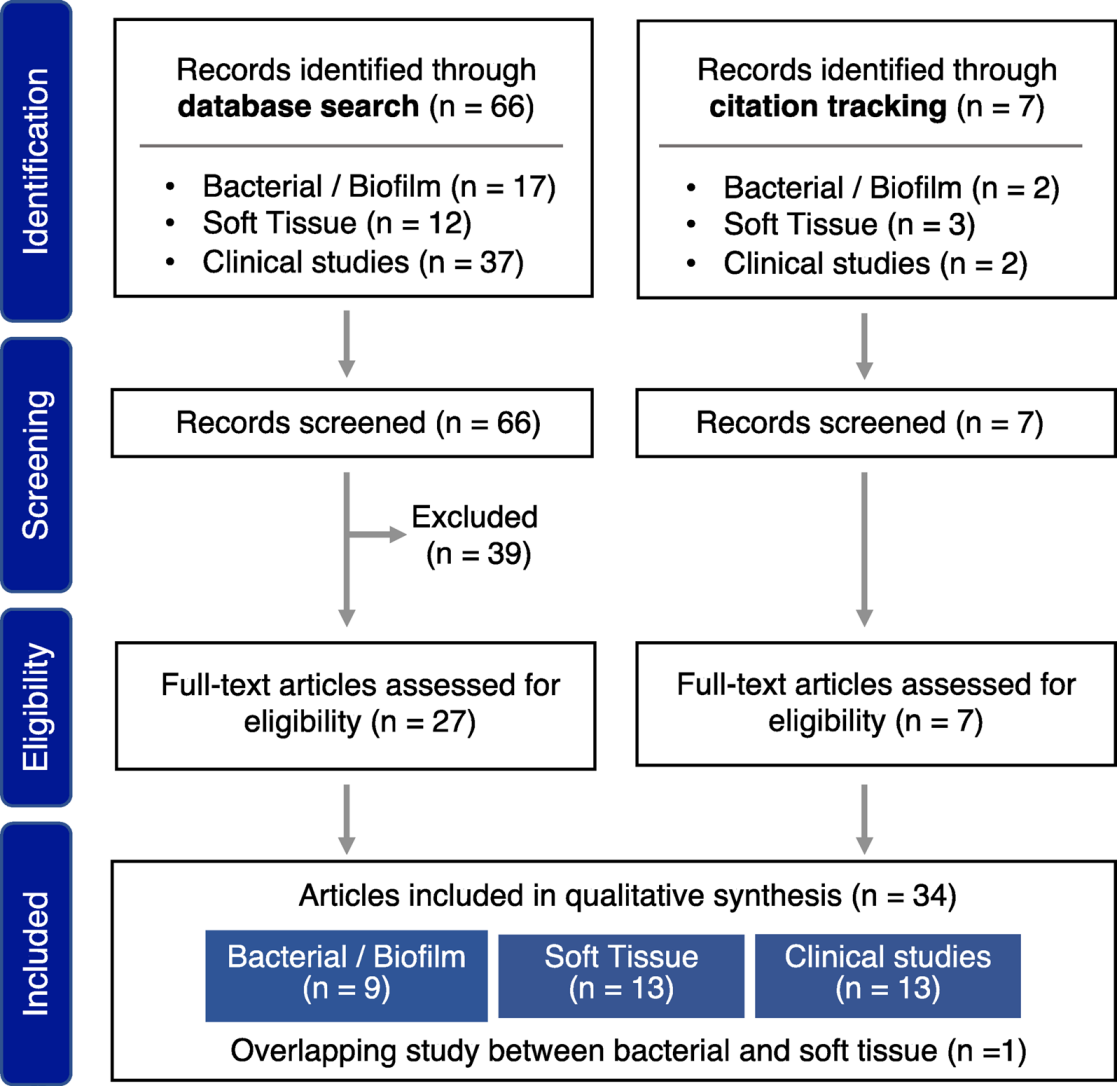
PRISMA flow diagram of study identification, screening, and inclusion. PubMed searches (PICO-based, outcome-specific queries) retrieved 66 records for full-text assessment, of which 27 studies met eligibility criteria for qualitative synthesis. An additional 7 eligible studies were identified through citation tracking. In total, 34 studies were included: 9 bacterial/biofilm, 13 soft-tissue (with 1 overlap), and 13 clinical studies

Study selection was performed in two stages: (1) title/abstract search/screening and (2) full-text evaluation for final eligibility. Three reviewers independently screened titles/abstracts and full texts for study selection and eligibility, with disagreements resolved by discussion and consensus. After full-text assessment of 66 searched records, 27 studies were included in the qualitative synthesis, comprising 7 bacterial/biofilm, 10 soft-tissue (1 overlapping between bacterial and soft-tissue), and 11 clinical studies. In addition to database-identified studies, 7 (2 bacterial/biofilm, 3 soft-tissue, and 2 clinical studies) articles were incorporated through manual citation tracking. Finally, a total of 34 articles, including 9 bacterial/biofilm, 13 soft-tissue (1 overlapping between bacterial and soft-tissue), and 13 clinical studies were selected (Fig. 6).

#### Data extraction and synthesis

Data were extracted into a standardized spreadsheet. Extracted variables included (as reported): study design/model (in vitro/animal/human), substrate (titanium/zirconia; implant/abutment), surface type/topography, UV protocol (wavelength band, exposure duration, device configuration, timing relative to use), comparator condition, and outcome measures relevant to the bacterial/biofilm, soft-tissue, and clinical domains. Given substantial heterogeneity in UV protocols, surface systems, study designs, and outcome measures, formal meta-analysis was not considered appropriate. Instead, findings were synthesized qualitatively using a domain-based narrative framework focused on bacterial/biofilm behavior, soft-tissue biology, and clinical peri-implant outcomes.

Because the included studies encompassed heterogeneous in vitro, animal, and clinical designs with substantial variability in methodologies and endpoints, a formal unified risk-of-bias assessment was not performed. Instead, study limitations, methodological variability, and consistency of findings were qualitatively considered during synthesis and interpretation. The review protocol was not prospectively registered.

### Antibacterial effects of UV photofunctionalization on titanium surfaces

#### Evidence base and experimental settings

The antibacterial evidence in the included literature is dominated by in vitro titanium–microbe assays, complemented by an in vivo human oral-cavity exposure study using orthodontic miniscrews [120], as summarized in Table 1. Across experimental systems, outcomes are commonly reported as initial bacterial attachment, biofilm coverage/biomass, biofilm thickness/architecture, and, less consistently, viability-related measures [121].

**Table 1:** UV photofunctionalization—bacterial outcomes

References	First author	Material	UV protocol	Key findings
[21]	Gallardo-Moreno (2009)	Polished Ti6Al4V alloy	UV (257.7 nm, 2.6 mW/cm^2^) for 15 h	UV treatment significantly reduced initial adhesion rates of S. aureus and S. epidermidis and decreased the number of bacteria retained after exposure to shear forces
[71]	Yamada (2014)	Titanium (mirror-polished, turned, acid-etched, or shot-blasted)	UVA (352 nm) or UVC (254 nm) at 3.0 mW/cm^2^ for 48 h	UVC irradiation reduced attachment and biofilm formation of S. aureus and S. pyogenes regardless of surface topography, showing superior results to UVA on micro-roughened surfaces
[23]	Avila (2015)	Machined commercially pure titanium	12 min UV via proprietary photo device (TheraBeam Super Osseo)	Photofunctionalization reduced initial attachment of human polymicrobial oral communities by 2.6-fold and subsequent biofilm formation by 3-fold. Bacterial community profiles appeared different between UV-treated and untreated titanium in the initial attachment phase
[22]	Zhang (2017)	Alkali-treated titanium with nanonetwork structures (TNS)	High-intensity UVC (254 nm, 100 mW/cm^2^) for 15 min	UV-TNS reduced colonization by Actinomyces oris during initial attachment and inhibited biofilm formation for up to 6 h, while enhancing osteoconductive profiles of bone marrow mesenchymal stem cells
[121]	Jain (2018)	Anodized titanium (amorphous, anatase, rutile, or mixed phases)	UVA (365 nm) or UVC (254 nm) for 10 min	UVA pre-irradiation achieved at least 20% reduction in S. sanguinis attachment; anodized layers with primarily anatase or mixed phases showed > 50% killing efficacy
[24]	Ishijima (2019)	Machined commercially pure titanium	12 min UV via proprietary photo device (TheraBeam Super Osseo)	UV treatment suppressed human oral bacterial attachment for at least 7 days; biofilm thickness remained under 8 μm on UV surfaces vs. 16 μm on untreated ones
[123]	Dini (2020)	Machined cpTi and Plasma Electrolytic Oxidation (PEO) treated Ti	UVC (253.7 nm, 2.34 mW/cm^2^) for 96 h	UV-mediated photofunctionalization reduced initial S. sanguinis adhesion at 1 h and enhanced blood plasma protein adsorption
[122]	Wen (2023)	Smooth and anodized nano-engineered titanium	UVC (253.7 nm, 107 µW/cm^2^) for 24 h	Both smooth and nano-surfaces effectively inhibited P. gingivalis adhesion after irradiation, with smooth surfaces showing improved fibroblast response
[120]	Goel (2024)	Titanium temporary anchorage devices (miniscrews)	UVA (350 nm) and UVC (250 nm) for 15 min in a calibrated chamber	In vivo study showed a significant reduction of S. sanguinis colonies on UV-treated miniscrews compared to untreated controls after 6 months

####  Early-phase bacterial attachment inhibition

Across multiple in vitro models, UV photofunctionalization most consistently reduced early bacterial adhesion to titanium. In a controlled in vitro attachment/biofilm study using UVA or UVC pre-irradiation, metabolically active adherent bacterial area, particularly Staphylococcus aureus or Streptococcus pyogenes, was reduced on both polished and turned titanium, with UVC frequently producing the lowest coverage [71]. The inhibitory effect was also observed on micro-roughened titanium; descriptive SEM assessments emphasized fewer bacteria retained within micro-pits after UV exposure [71]. Consistent reductions in early attachment have also been reported on anodized titanium oxide. In one in vitro study, UVA pre-irradiation produced a trend of ≥ 20% reduction in bacterial attachment across different oxide crystallinity groups, indicating an attachment-inhibitory effect that can extend to oxide-modified surfaces [121].

#### Biofilm formation and maturation outcomes

Beyond initial adhesion, multiple studies have reported that UV-treated titanium suppresses downstream biofilm development. In a saliva-derived, multi-species oral community model, UV-treated machined titanium showed reduced oral bacterial attachment and biofilm formation, while overall bacterial viability was not decreased—consistent with an anti-adhesive effect rather than a broadly bactericidal mechanism [23]. UV has also been associated with improved biofilm structural outcomes. In one study, untreated titanium developed biofilms with thicknesses on the order of ~ 16 μm by day 7, whereas UV-treated surfaces remained below ~ 8 μm [24]. The same report noted a larger biofilm-driven increase in apparent surface roughness on untreated titanium compared with UV-treated titanium, suggesting a potential feed-forward “vicious cycle” in which biofilm accumulation further increases plaque-retentive microtexture and thereby promotes additional bacterial retention [24].

#### UV spectrum dependence and associated physicochemical changes

Comparative UVA vs. UVC experiments indicate that antibacterial outcomes occur alongside strong physicochemical activation of titanium surfaces. Following UVA exposure, water contact angles were reduced to approximately 20–30°, whereas UVC reduced contact angles to ~ 0–15° across tested titanium surfaces [71]. In the same work, surface carbon decreased after UVA and more prominently after UVC, supporting the concept that hydrocarbon removal is a major component of UV-driven surface activation relevant to bacterial inhibition. Consistent with these physicochemical differences, UVA and UVC both reduced adherent bacterial signals, though differences between UVA and UVC were not uniformly significant across all surfaces and timepoints [71].

#### Surface dependence and mechanistic heterogeneity

Because bacterial attachment is a primary concern at the transmucosal abutment and crown-margin region, most antibacterial photofunctionalization studies have used machined or polished (smooth) titanium surfaces as clinically relevant proxies for abutment finishes [23, 24, 71]. A smaller subset has examined roughened or textured surfaces to reflect implant fixture topographies [71, 121]. Overall, UV photofunctionalization has shown inhibitory effects across a range of surface textures and bacterial models [71], although the magnitude of benefit can vary with UV source and dose (wavelength/exposure time), time point of assessment, and bacterial species.

Most selected studies evaluated commercially pure (cp.) titanium; however, reduced adhesion of Staphylococcus aureus and Staphylococcus epidermidis has also been demonstrated on UVC-treated Ti–6Al–4 V alloy [21]. With respect to bacterial taxa, inhibitory effects have been reported for Staphylococcus aureus and Streptococcus pyogenes on polished, machined, and microrough titanium surfaces [71]. UVC-treated nanofeatured titanium has been shown to reduce adhesion of key peri-implant taxa including Porphyromonas gingivalis [122] and Actinomyces oris [22]. In a separate in vitro study using plasma electrolytic oxidized titanium, UV treatment significantly reduced Streptococcus sanguinis adhesion [123]. Importantly, beyond single-species assays, saliva-derived oral community models demonstrated that collective bacterial attachment and biofilm formation were suppressed on UV-treated machined titanium [23, 24], supporting relevance to clinically realistic microbial consortia.

#### In vivo human oral-cavity exposure evidence

Translational support is further provided by a clinical exposure study in which orthodontic miniscrews were placed intraorally and retrieved for microbial quantification after six months. UV-treated devices exhibited a substantially lower Streptococcus sanguinis burden than controls (approximately one-quarter of the control level) [120].

#### Summary of antibacterial evidence

**Fig. 7:**
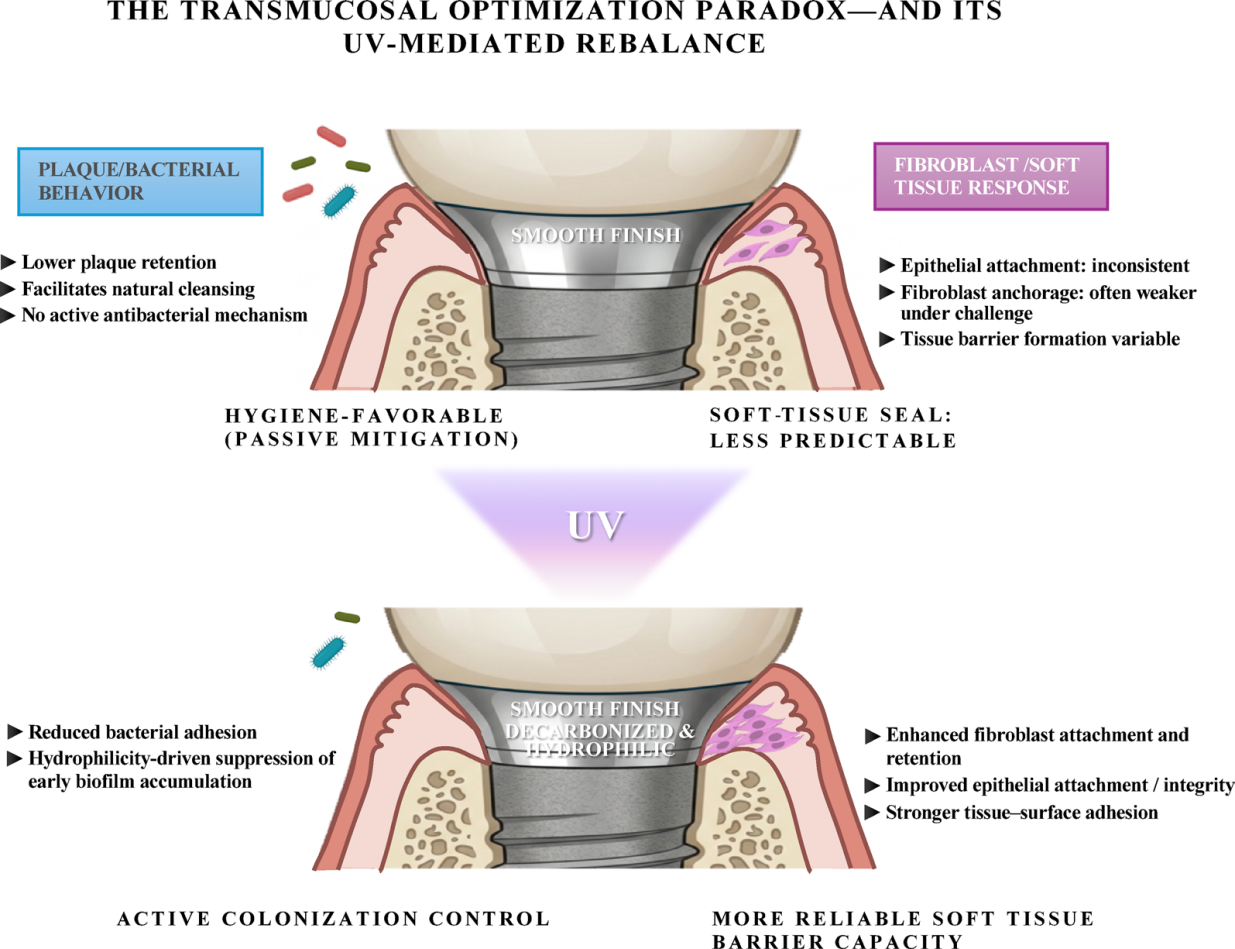
The transmucosal optimization paradox and its UV-mediated rebalance. Summary schematic of the systematic review findings across bacterial and soft-tissue studies, illustrating how UV photofunctionalization can shift the balance of competing design pressures at the transmucosal implant abutment interface. The central paradox is that hygiene-favorable smoothness can reduce plaque retention yet leave soft-tissue barrier performance less predictable; UV photofunctionalization is proposed to rebalance this toward both plaque control and barrier formation. Top row (as-received, smooth finish): polished or smooth transmucosal surfaces are generally hygiene-favorable because they reduce plaque retention and facilitate passive mitigation through natural cleansing and routine oral hygiene. However, barrier-related biology can be variable, with less predictable epithelial attachment, fibroblast anchorage, and overall soft-tissue sealing capacity. Bottom row (UV-photofunctionalized): UV treatment restores a high-energy, hydrophilic surface state and reduces initial bacterial adhesion and early biofilm accumulation, indicating an “active” interface-level suppression of colonization beyond morphology-driven plaque control. In parallel, UV photofunctionalization enhances soft-tissue cell attachment and retention—supporting improved epithelial integrity, stronger connective-tissue adhesion, and newly acquired or strengthened soft-tissue barrier capacity. Collectively, the figure illustrates an interface-first concept in which UV photofunctionalization complements smooth-surface hygiene advantages by simultaneously improving both early colonization control and peri-implant mucosal barrier biology

Collectively, the bacterial-focused literature indicates that UV photofunctionalization reduces early bacterial attachment on titanium across multiple surface types and experimental models, with additional evidence for suppressed biofilm development and reduced biofilm thickness (Fig. 7). These outcomes typically coincide with physicochemical surface activation—most notably superhydrophilicity and decarbonization—changes that are generally more pronounced after UVC than UVA exposure. The inhibitory effect has been observed across a range of bacterial taxa tested, from early colonizers to peri-implant–relevant species and saliva-derived communities. At the same time, antibacterial effects are not uniform: surface chemistry, topography, and oxide structure can modulate the magnitude of the response, underscoring mechanistic heterogeneity. Importantly, translational evidence extends beyond bench models: in vivo oral-cavity exposure data demonstrate a reduced burden of an early colonizer on UV-treated orthodontic miniscrews after six months, supporting real-mouth relevance and durability of the anti-adhesive effect.

### Soft-tissue cell responses to UV photofunctionalization

#### Biological premise: peri-implant health is an interface problem

Compared with the tooth–periodontium attachment apparatus, peri-implant connective tissue is structurally more vulnerable and exhibits limited direct insertion into abutment surfaces, creating a permissive corridor for sulcular microbial accumulation and apical spread. Because many strategies intended to strengthen connective-tissue attachment rely on surface texturing—which can also increase plaque retention—approaches that enhance soft-tissue affinity without altering topography are especially relevant (see Fig. 4).

UV photofunctionalization addresses this “interface-first” need by reversing titanium surface aging. Importantly, unlike osseointegration, the field still lacks consensus on whether smoother or rougher transmucosal surfaces are intrinsically superior for establishing a durable mucosal barrier. This incomplete baseline framework makes rational optimization of the soft-tissue seal more challenging. Accordingly, the focus of this section is not to adjudicate surface-topography design for the transmucosal zone, but to summarize evidence for UV-driven biological enhancement (Table 2). Reflecting translational relevance to abutments and prosthetic components, most studies have used machined (smooth) surfaces when testing UV effects on titanium [124–126] and zirconia [102, 127].

**Table 2:** UV photofunctionalization—soft tissue outcomes

References	First Author	Material	UV Protocol	Key Findings
[26]	Hoshi (2010)	CP Titanium (grade 2) coated with TiO_2_ film (1–3 μm)	24 h UV irradiation (368 nm)	Proliferation of human periodontal ligament fibroblasts significantly increased on TiO2-coated disks compared to uncoated controls; the effect was dependent on film thickness
[127]	Yang (2015)	Zirconia (smooth polished or rough air-abraded)	24 h UV light (250 ± 20 nm)	UV treatment on rough zirconia significantly enhanced 24-h human gingival fibroblast (HGF) adhesion, proliferation, and collagen release. Smooth surfaces showed improved initial 3-h adhesion
[25]	Areid (2018)	Nanostructured titanium (TiO2 sol-gel or hydrothermal coatings)	15 min UV light (254 nm)	UV light treatment significantly enhanced surface hydrophilicity. Nanostructured coatings (MA and HT) improved HGF adhesion resistance and sustained proliferation for up to 10 days
[129]	Okubo (2020)	Polished grade 2 titanium	12 min UV light (TheraBeam Super Osseo)	UV treatment reversed the compromised biological capability caused by polishing, doubling epithelial cell attachment counts compared to machined surfaces
[128]	Nakhaei (2021)	Machined grade 2 titanium	12 min UV light (TheraBeam Super Osseo)	UV treatment enhanced the attachment, spreading, and retention of human oral epithelial cells via surface decarbonization. Hemi-desmosome-related molecules, integrin beta 4 and laminin-5, were upregulated at the gene and protein levels in the cells on UV-treated surfaces
[131]	Razali (2021)	Zirconia (YSZ, ATZ) and grade 2 titanium	12 min UV light (TheraBeam Super Osseo)	Photofunctionalization significantly improved the biological seal of the peri-implant mucosal interface, with YSZ showing the lowest permeability to tritiated water
[130]	Ikeda (2021)	Smooth machined titanium	15 min UV light (TheraBeam Affiny)	UV treatment increased the adhesion strength of oral connective tissue by six times and doubled tissue retention time on the surface
[132]	Rutkunas (2022)	High-translucent and ultra-translucent multi-layered zirconia	48 h UVC light (253.7 nm, 3.49 mW/cm^2^)	UV surface photofunctionalization altered HGF viability and produced favorable results for cell proliferation
[122]	Wen (2023)	Smooth and anodized nano-engineered titanium	24 h UVC lamp (253.7 nm, 107 µW/cm^2^)	UV treatment enhanced human gingival fibroblast (HGF) adhesion and proliferation on smooth surfaces while effectively inhibiting P. gingivalis adhesion
[102]	Suzumura (2023)	Milled and glazed (silica-surfaced) zirconia	1 min Vacuum UV (VUV, 172 nm)	VUV photofunctionalization induced specimen-to-specimen transmigration of human oral fibroblasts. The bioactivated surfaces sustained these biological effects for at least 7 days
[126]	Matsuura (2025)	Machined grade 4 titanium	1 min Vacuum UV (VUV, 172 nm)	VUV treatment doubled fibroblast attachment and proliferation, mitigated cellular oxidative stress (reduced ROS by 50%), and significantly improved cell migration
[124]	Komatsu (2025a)	Titanium implant abutments	1 min Vacuum UV (VUV, 172 nm)	VUV treatment led to a 3.9-fold increase in fibroblast attachment and nearly complete collagen coverage on challenging axial abutment surfaces
[125]	Komatsu (2025b)	Titanium healing abutments	1 min Vacuum UV (VUV, 172 nm)	Decarbonization via VUV enhanced fibroblast attachment (2–4 times) and proliferation (2–6 times) by fostering homeostatic cellular phenotypes involving proteoglycan/GAG interactions and antioxidant defense

#### Connective tissue fibroblasts: enhanced attachment, growth, and retention on photofunctionalized titanium

Across in vitro models, photofunctionalization consistently increases early fibroblast attachment and subsequent growth on titanium (Table 2). In a healing-abutment model (representative prosthodontic components), 1-minute vacuum UV (VUV) treatment (172 nm) rendered titanium hydrophilic, resulting in improved attachment, proliferation, and retention of human gingival fibroblasts [124].

In a related mechanistic study using human gingival connective tissue fibroblasts, 1-minute UV treatment reduced surface carbon substantially (from ~ 60 to ~ 29%) without changing topography, and increased fibroblast attachment by ~ 2–4-fold, proliferation by ~ 2–6-fold, and retention against dislodging forces by ~ 2–5-fold [125]. Transcriptomic profiling further supported a phenotype compatible with tissue stabilization: extracellular matrix production and proteoglycan/glycosaminoglycan (GAG)-binding pathways were upregulated, while inflammatory/immune-response gene sets were downregulated on UV-treated titanium [125]. The same work also provided functional support for a GAG-dependent adhesion mechanism—enhanced adhesion was negated by enzymatic cleavage of GAGs, confirming its decisive role.

A clinically relevant nuance highlighted by abutment-shaped specimens is the combined importance of geometry and fluid behavior at the interface. UV treatment improved medium wetting and coverage around titanium surfaces and supported stronger cell recruitment even on curved or anatomically challenging regions—areas where untreated surfaces may remain partially unwetted and therefore “geographically unfavorable” for initial cell colonization [125].

Consistent with these observations, an independent study also reported increased attachment and proliferation of human gingival fibroblasts on UVC-treated titanium [122]. Finally, enhancement of human periodontal ligament fibroblast responses has also been reported on titanium with a controlled TiO_2_-coated layer [26]. Notably, the magnitude of enhancement correlated with TiO_2_ thickness, providing mechanistic support that the UV response is mediated through the oxide layer and is not merely a nonspecific “cleaning” phenomenon.

#### Collagen layer formation and “seal-like” coverage: toward a connective tissue barrier phenotype

Beyond increasing cell number, several studies emphasize the quality and continuity of the deposited extracellular matrix on the abutment surface. In the healing-abutment model, UV-treated surfaces achieved early, uniform collagen layer coverage, whereas untreated abutments exhibited insufficient collagen continuity that persisted with time [124]. These observations also align with transcriptome-level enrichment of extracellular matrix and protease inhibition pathways on UV-treated titanium, suggesting not only increased matrix formation but improved protection from proteolytic degradation [125].

#### Resistance to detachment under mechanical and chemical challenges

Functional sealing must withstand daily mechanical and biochemical insults (mastication, brushing, instrumentation, and protease-rich inflammatory conditions). Detachment testing on abutment surfaces demonstrated pronounced differences in adhesion robustness: under strong mechanical stimulus, nearly all cells detached from untreated abutments, whereas UV-treated abutments retained a measurable fraction of cells after detachment testing (15.9% ± 4.8% remaining) [124]. Under strong chemical challenge, approximately 80% of cells detached from untreated abutments, while UV-treated surfaces showed substantially higher resistance (79.1% ± 21.0% cells remaining) [124]. When expressed as retention enhancement, resistance to mechanical and chemical detachment increased 11.3- and 4.3-fold, respectively, after UV photofunctionalization. Enhanced cell retention following detachment challenges has also been reported in an independent study, supporting reproducibility across experimental settings [25].

#### Redox balance and inflammatory tone: a stress-mitigating phenotype in gingival fibroblasts

Early wound healing around implants involves oxidative stress, and excessive oxidative burden can impair fibroblast function and migration. In a cell stress model, human gingival fibroblasts on UV-treated titanium showed reduced intracellular reactive oxygen species (ROS) (~ 50% lower than controls) and preserved intracellular antioxidant capacity, including maintenance of glutathione reserves under oxidative challenge [126]. In the same study, expression of IL-1β and IL-8 decreased by ~ 40–60% on UV-treated titanium. Concordantly, the mechanistic transcriptomic study on human gingival connective tissue fibroblasts reported enhanced redox balance (glutathione reserve/maintenance during oxidative stress) and downregulation of immune/inflammatory programs on VUV-treated titanium [125]. Together, these data support a model in which photofunctionalization promotes not only quantitative growth but also a homeostatic, stress-resistant fibroblast phenotype favorable for early seal formation.

#### Cell migration and “interface bridging”: transmigration as a functional readout

Migration/spreading across interface discontinuities is clinically relevant because microgaps exist at the implant–abutment–prosthesis complex and within restoration margins. A plate-to-plate assay using human gingival fibroblasts showed that UV-treated titanium enhanced recruitment and transmigration across a gap and preserved migratory capacity even when fibroblasts were functionally impaired by oxidative stress [126]. Quantitatively, the leading zone extended (~ 20% longer on UV-treated titanium), and the “volume zone” of migrated fibroblasts was more than doubled compared with untreated controls; notably, severely damaged fibroblasts that failed to migrate on untreated titanium could migrate/spread on UV-treated titanium [126].

A complementary material-expansion study reported horizontal transmigration of human oral fibroblasts exclusively on VUV-treated zirconia surfaces, including migration across a 150-µm specimen gap [102]. The same work reported increased early attachment, higher cell density, increased BrdU incorporation, and increased collagen deposition on VUV-treated zirconia—while noting minimal changes in gene expression between treated and control groups [102]. Collectively, these transmigration models provide a functional framework for “interface bridging” as an experimentally tractable surrogate phenotype for seal formation at imperfect interfaces of implant abutment- and restoration-relevant materials of titanium and zirconia.

#### Epithelial cells: recovery of attachment competence on compromised titanium surfaces

Because the peri-implant seal is composite (epithelial + connective tissue), epithelial attachment behaviors represent an essential parallel axis. In a study using human oral epithelial cells, UV treatment increased attachment, adhesion strength, and retention on titanium [128]. Clinically, transmucosal surfaces are frequently altered by polishing, scaling, or other instrumentation during maintenance, and such procedures can introduce chemical contamination or otherwise compromise cell–surface interactions. Consistent with this concern, an epithelial model showed that polishing-induced contamination reduced epithelial attachment to titanium, whereas UV treatment restored attachment competence [129]. Together, these findings support photofunctionalization not only as an adjunct at initial prosthetic placement, but also as a potential reconditioning (“re-entry”) strategy when abutment surfaces have been modified during maintenance care or peri-implantitis management.

#### Soft-tissue adhesion/retention models: toward clinically interpretable endpoints

To bridge reductionist cell assays to clinically meaningful peri-implant sealing, experimental models that directly assess tissue-level adhesion and retention to titanium are particularly informative. In an ex vivo–style model using keratinized oral mucosa connective tissue, UV photofunctionalization markedly enhanced both the durability and strength of tissue attachment to titanium surfaces [130]. Specifically, tissue sections placed on UV-treated titanium remained adherent for more than twice as long under continuous mechanical agitation compared with untreated controls (mean retention ~ 15.5 h vs. ~ 7.5 h) [130]. This is not merely a modest shift in average behavior: nearly all tissue samples on UV-treated titanium remained attached beyond early time points, whereas approximately half of the control samples detached within the first few hours, indicating a qualitative improvement in interfacial stability rather than a marginal effect. Beyond time-to-detachment, the same study quantified adhesion strength using tensile testing and demonstrated an approximately six-fold increase in the force required to detach mucosal connective tissue from UV-treated titanium compared with untreated surfaces [130].

A complementary and conceptually important advance is provided by a three-dimensional organotypic peri-implant mucosal model that directly quantified the quality of the soft-tissue seal formed against photofunctionalized abutment materials [131]. In this model, primary human gingival keratinocytes and fibroblasts were co-cultured on an acellular dermal matrix to reconstruct a stratified peri-implant mucosa, and zirconia and titanium specimens—either untreated or UV treated—were inserted into the tissue construct. The integrity of the mucosal barrier was then quantified by measuring the permeability of a tracer (tritiated water) across the material–tissue interface, providing a functional readout of “biological seal” quality rather than a purely morphological endpoint. Photofunctionalization significantly reduced tracer penetration compared with untreated controls, indicating a tighter and more effective epithelial–connective tissue seal at the interface. Histological analysis further showed more organized epithelial stratification and less interface disruption on UV-treated surfaces, supporting the interpretation that surface photofunctionalization improves not only initial cell attachment but also the structural coherence of the peri-implant mucosal barrier.

#### Material and UV considerations and broader surface-biology context

The enhanced attachment and proliferation of soft-tissue cells observed after photofunctionalization can be generalized on titanium and zirconia, based on the studies selected here [127, 132]. However, the magnitude and consistency of this enhancement depend on several interacting factors, including the base material, its surface state and preconditioning (e.g., storage-related biological aging or prior surface treatments) [31], and, critically, the wavelength and intensity of the UV source.

Comparative evidence suggests that vacuum ultraviolet (VUV; 172 nm) can elicit stronger biological effects than conventional UVC on both titanium and zirconia. This is exemplified by plate-to-plate transmigration—an aggressive and highly discriminative endpoint that is rarely observed on untreated substrates—reported after VUV activation [102, 126]. Exposure time further modulates the response: with VUV, biologically meaningful activation can be achieved within one minute, whereas lower-energy UV sources typically require longer irradiation to reach comparable surface activation [29, 30].

From a surface–biology perspective, these findings support the notion that the key driver is not simply “increased hydrophilicity,” but the conversion to a superhydrophilic, low-carbon surface state that enables reliable and robust cell–surface interactions. Across multiple studies, effective photofunctionalization is associated with a marked reduction in surface carbon contamination (often to < 15 at%) [5, 6, 82, 107].

#### Soft-tissue interface summary

Across the papers included, UV photofunctionalization (including vacuum ultraviolet, VUV) was consistently positioned as an interface-first strategy to strengthen peri-implant mucosal defense by improving early cell/tissue engagement and resistance to detachment (Fig. 7). A central mechanistic theme was that UV treatment reverses “biologic aging”–type degradation by restoring hydrophilicity and reducing surface hydrocarbons, thereby facilitating more stable cell–surface interactions without altering surface morphology.

Functionally, the soft-tissue response literature in this set emphasized two outcomes that are particularly relevant to peri-implant health:

1. Retention/adhesion under challenge (agitation, tensile pull, trypsinization, strong mechanical stimulation), and
2. Barrier-building behaviors (enhanced recruitment/spreading, migration/transmigration across discontinuities, and epithelial barrier performance metrics such as permeability).

In an ex vivo rat palatal keratinized mucosa model, UV treatment increased tissue-section retention time and markedly increased tensile adhesion strength. Complementing this tissue-retention approach, a three-dimensional organotypic peri-implant mucosal model using human gingival keratinocytes and fibroblasts demonstrated that photofunctionalization significantly reduced trans-interface permeability, indicating formation of a tighter and more functional soft-tissue seal.

In VUV-focused models designed to mimic clinical stressors (e.g., abutment exchange, oxidative stress), UV-treated titanium supported fibroblast recruitment and transmigration, including migration by oxidatively damaged fibroblasts that failed to migrate onto untreated surfaces. Evidence also extended beyond titanium to zirconia/zirconia-containing systems, where UV-associated improvements in cytocompatibility, adhesion-related protein expression, and transmigration/barrier-relevant responses were reported. Collectively, these studies support framing UV photofunctionalization—within the soft-tissue domain—as a peri-implant mucosal defense technology.

### Clinical evidence linking UV photofunctionalization to peri-implant health

#### Scope of available clinical evidence and endpoints

Across the 13 included papers (Table 3), the clinical evidence base comprises randomized/controlled clinical trials and prospective cohorts [133–139], retrospective/case-control clinical studies [140–143], and a case series [144]. In addition, a large retrospective clinical analysis specifically examined early implant failure as a peri-implant health–relevant endpoint and identified photofunctionalization as a significant independent factor reducing early failure risk [143]. Collectively, the dominant clinical endpoints have been osseointegration-linked stability development—most commonly resonance frequency analysis (RFA) expressed as implant stability quotient (ISQ) and derivative indices reflecting rate of stability gain (e.g., “osseointegration speed index,” OSI: ISQ increase per month) [140, 142, 145]—and radiographic peri-implant bone measures including marginal bone level (MBL)/marginal bone loss and, in some studies, radiographic bone microarchitecture surrogates [133, 137]. A smaller subset of papers reported peri-implant soft-tissue and esthetic outcomes [133, 134], while postoperative morbidity and complications were variably documented, particularly in the case-series context [144].

**Table 3:** UV photofunctionalization—clinical outcomes

References	First author	Material	UV protocol	Key findings
[145]	Suzuki (2013)	Titanium (oxidation-treated, TiUnite)	15 min UV light (TheraBeam Affiny)	Prospective Study: UV treatment eliminated the “stability dip” typically observed during the first 4 weeks of healing. ISQ values either remained constant or increased during early healing. The average ISQ at Week 6 reached 78.0. The OSI (ISQ increase/month) for photofunctionalized implants was 6.3 and 3.1 when their initial ISQ was 65 to 70 and 71 to 75, respectively, whereas the OSI values for as-received implants calculated from literature ranged from − 3.0 to 1.17 with an average of − 0.10
[144]	Funato (2013a)	Titanium (microroughened, Osseotite Certain)	15 min UV light (TheraBeam Affiny)	Case Series: High success rates were achieved in compromised bone sites (fresh extraction sockets, sinus elevation, and vertically augmented bone) with early loading protocols of 2.1 to 4.5 months. Demonstrated a significant gain in marginal bone level, increasing from − 0.35 mm at loading to + 0.16 mm at 1 year. Bone often grew coronal to the implant platform
[140]	Funato (2013b)	Titanium (microroughened, Osseotite Certain)	15 min UV light (TheraBeam Affiny)	Retrospective Study: Success rate of 97.6% (vs. 96.3% untreated) with healing time reduced to 3.2 months (vs. 6.5 months). OSI ranged from 2.0 to 8.7, being highest (8.7 ± 4.1) for low primary stability (initial ISQ: 40–49). Compared with literature, ISQ increase (10.7–26.2) and OSI (2.0–8.7) were substantially higher than reported values (range − 5.0 to 4.6 and − 1.8 to 2.8, respectively)
[141]	Hirota (2016)	Titanium (TiUnite and NobelActive)	15 min UV light (TheraBeam Affiny)	Case-Control Study: Average OSI was significantly higher for UV implants (3.7 ± 2.9) than for as-received (0.0 ± 1.0). The OSI in complex cases was 4.2 ± 3.2 for UV implants and 0.2 ± 0.9 for as-received implants. The OSI with simultaneous sinus elevation was 5.5 ± 3.5 for UV implants and 0.2 ± 1.1 for as-received implants
[142]	Kitajima (2016)	Titanium (microroughened, Osseotite Certain)	15 min UV light (TheraBeam Affiny)	Retrospective study: implants with extremely low primary stability (ISQ < 60) were successfully stabilized, with all implants reaching a high mean ISQ of 74.3 at second-stage surgery. Among implants with baseline ISQ values of 55 or lower, the OSI ranged from 3.9 to 4.7, considerably higher than the OSI values reported for as-received implants in the literature (0.36–2.8)
[143]	Hirota (2018)	Titanium (Nobel Biocare machine or rough surface)	15 min UV light (TheraBeam Affiny)	Retrospective Study: Analyzed 563 implants; photofunctionalization significantly reduced early failure risk (OR = 0.30). Failure rate: 1.3% (UV) vs. 4.3% (untreated)
[139]	Puisys (2020)	Titanium (BioHorizons Tapered Plus)	12 min UV light (TheraBeam SuperOsseo)	Triple-Blinded Split-Mouth RCT: UV treatment significantly increased removal torque (RT) values at 2, 3, 4, and 8 weeks, indicating enhanced early osseointegration and mechanical stability
[136]	Hirota (2020)	Titanium (Brånemark Groovy and NobelReplace)	15 min UV light (TheraBeam Affiny)	Prospective Study (7-Year Follow-up): Reported 100% success rate for regular and complex cases. Mean ISQ values in complex cases increased by 21.9 points during healing
[133]	Shah (2021)	Tapered internal implants (Alpha Dent Active)	20 min UV light (SK Dent UV chamber)	Randomized Controlled Trial (RCT): In immediate placement, UV-pretreated implants showed significantly higher stability at all intervals from 2 weeks to 12 months. The 12-month ISQ being 72.08 ± 1.38 for UV implants and 65.09 ± 1.76 for control implants, demonstrating that photofunctionalization both accelerates the healing process and increases the absolute magnitude of final osseointegration
[134]	Choi (2021)	Titanium (SLA-treated, Osstem TS IV)	15 min UV light (TheraBeam Affiny)	Randomized Double-Blinded RCT: UV treatment significantly enhanced implant stability at week 4 and 4 months of healing in the posterior maxilla with poor bone quality. UV treatment also reduced early marginal bone loss at week 4 in type 2 bone
[135]	Farsiani (2025)	Titanium (Gravis Dental Implants)	10 s UV light (Dentis UV device)	Split-Mouth RCT: Fractal Dimension (FD) analysis showed a statistically significant increase in bone microstructure quality and complexity around UV-treated implants
[137]	Murali Krishna (2025)	Titanium (tapered root form, DIO implants)	20 s VUV light (DIO UV Activator)	Split-Mouth RCT: In controlled diabetic patients, UV-treated implants showed significantly higher ISQ increase (4.4 ± 1.89 vs. 2.6 ± 1.17) after 3 months and significantly less distal crestal bone loss at 9 months
[138]	Pathak (2025)	Titanium (DIO UV Active implants)	20 s VUV light (DIO UV Activator 2)	Prospective RCT: UV protocol significantly increased secondary stability (73.4 ± 9.5 vs. 61.2 ± 4.4) and OSI (7.2 ± 3.3 vs. 2.3 ± 2.0) at 3 months. No “stability dip” was observed in the UV group

In the available evidence, peri-implant health is most directly supported when accelerated osseointegration (greater and/or earlier stability development) is accompanied by (i) improved or comparable peri-implant bone maintenance (MBL), and/or (ii) stable peri-implant mucosal parameters and low inflammatory complications. Importantly, the early-failure study provides a complementary and clinically immediate peri-implant health perspective by showing that photofunctionalization significantly reduced the odds of early implant failure in a multivariate model [143]. The evidence that explicitly connects these domains remains limited, but several studies provide clinically relevant “interface-first” signals—early stability gains together with favorable peri-implant tissue outcomes—particularly in accelerated protocols or complex host/site conditions [133, 140, 142] and in cohorts enriched for site-development procedures (e.g., simultaneous guided bone regeneration, sinus elevation, or fresh extraction sockets) [140] and long-term complex-case follow-up [141]. Notably, the controlled-diabetes split-mouth trial extends the “interface-first” narrative to a medically compromised host context, pairing an early stability advantage with reduced crestal bone loss [137].

#### Early stability development as a clinical surrogate for accelerated osseointegration

In a prospective clinical cohort under an immediate-loading maxillary protocol, photofunctionalized implants achieved high ISQ values by week 6 and demonstrated comparatively high OSI values, with ISQ at week 6 around the upper 70s and OSI values reported as 6.3 and 3.1 depending on baseline ISQ strata [145]. These findings were interpreted as reflecting attenuation—or possible elimination—of the classic “stability dip,” with potential implications for enabling early loading protocols.

A large retrospective study evaluating accelerated protocols reported that the healing time before functional loading could be reduced from 6.5 to 3.2 months in complex cases, with maintained high success (97.6% reported for the photofunctionalized group), using ISQ-based monitoring as the primary quantitative indicator of osseointegration progress [140].

Complementing RFA-based stability metrics, a triple-blinded split-mouth randomized controlled clinical trial assessed early fixation using resistance to removal torque as a direct biomechanical surrogate of bone–implant anchorage [139]. In that study, UV “photo-activation” of titanium implants increased resistance to removal torque compared with paired non-treated controls, supporting an early healing-phase stability advantage consistent with accelerated secondary stability development.

Importantly, the osseointegration–peri-implant health linkage becomes most clinically meaningful in compromised conditions where early instability can predispose to micromotion, marginal bone loss, and inflammatory complications. In a clinical study focused on challenging and complex cases with low/very low/absent primary stability, photofunctionalized implants showed robust stability increases between placement and stage-two surgery; OSI as ISQ increase per month demonstrated that stability gains by photofunctionalization compensated for poor primary fixation, supporting expansion of implant indications in difficult hosts [142].

Consistent with this concept, a case-control clinical study comparing regular and complex indications (including simultaneous guided bone regeneration, sinus elevation, and fresh extraction sockets) reported substantially faster stability development with photofunctionalization. The average OSI was higher for photofunctionalized implants than untreated implants, and the difference was most pronounced in complex cases (OSI 4.2 ± 3.2 vs. 0.2 ± 0.9), including a sinus-elevation subgroup (OSI 5.5 ± 3.5 vs. 0.2 ± 1.1). Photofunctionalized implants also achieved ISQ at the second-stage surgery > 60 regardless of the severity of innate bone support, supporting a “stability catch-up” effect in compromised sites [141]. A 7-year prospective cohort further contextualized stability development by site category. Although complex cases exhibited markedly lower primary stability than regular cases, they showed a substantially larger ISQ increase by secondary surgery (+ 21.9 in complex vs. + 3.2 in regular), consistent with accelerated secondary stability development as a clinically observable feature of photofunctionalized implants in difficult sites [136].

More recently, a prospective randomized controlled trial tested the clinical feasibility of a short chairside protocol (20-second VUV, 172 nm) and still observed a clear “secondary stability advantage.” [138] While primary stability did not differ between groups at placement, secondary stability at 3 months and the OSI (monthly ISQ gain) were significantly higher in the photofunctionalized group, indicating that even very short exposure can translate into measurably faster stability development over time.

A randomized split-mouth trial in the posterior maxilla of controlled diabetic patients adds evidence that this “secondary stability advantage” can be observed even when systemic risk factors may constrain bone healing. The trial measured ISQ immediately after placement and at 3 months, and reported a significantly larger ISQ increase for photofunctionalized implants (mean change 4.4 ± 1.89) compared with non-photofunctionalized implants (2.6 ± 1.17), together with a higher OSI (1.42 ± 0.62 vs. 0.84 ± 0.39) [137].

#### Peri-implant marginal bone maintenance and radiographic bone quality

In a randomized double-blinded clinical trial focusing on the posterior maxilla, implants were either UV-treated or left untreated, and marginal bone levels were evaluated radiographically at 4 weeks, 4 months, and 1 year, with additional stratification by bone quality using CBCT grayscale values [134]. While overall marginal bone loss did not differ significantly between groups at most time points, a significant reduction in early bone loss at 4 weeks was observed in the UV-treated group within the better-quality (group II) bone subgroup. This finding suggests that photofunctionalization may exert its most detectable bone-preserving effect during the early remodeling phase.

In a split-mouth randomized clinical study, the effect of photofunctionalization on peri-implant bone quality was evaluated using fractal dimension analysis (FDA) of panoramic radiographs taken before and after implant placement [135]. Rather than focusing solely on linear MBL, this study aimed to capture changes in trabecular bone microarchitecture as a surrogate for bone quality and structural adaptation around the implant. The test (UV-treated) implants showed a statistically significant increase in fractal dimension values postoperatively, whereas the control group did not, indicating a more complex and mature trabecular pattern around photofunctionalized implants. Although this approach does not directly quantify marginal bone height changes, the results support the interpretation that UV treatment is associated with qualitative improvements in peri-implant bone structure, which may underlie more stable marginal bone behavior over time and complement conventional MBL measurements [135].

In a prospective split-mouth randomized clinical trial in controlled diabetic patients, UV-photofunctionalized and non-treated implants were compared with respect to implant stability, osseointegration speed, and crestal bone changes at 3 and 9 months [137]. The hypothesis that diabetes represents a systemic risk factor for impaired bone healing and increased peri-implant bone loss was tested. The UV-treated group demonstrated significantly less crestal bone loss, particularly on the distal aspect at 9 months, compared with the non-treated group.

In a randomized controlled clinical trial in the esthetic zone, immediate implants were placed after extraction and allocated to photofunctionalization (PF), platelet-rich plasma (PRP), or control pretreatment, with delayed loading and follow-up to 12 months [133]. Marginal bone loss (MBL) was assessed serially using standardized periapical radiographs and ImageJ-based measurements at 2, 4, 6, and 12 months. The study found that mean MBL increased over time in all groups, but no clinically or statistically significant difference in MBL was detected between the PF, PRP, and control groups, with values remaining within a clinically acceptable range. PF and PRP groups showed significantly higher implant stability values than the control group from 2 to 12 months, suggesting that photofunctionalization may not only accelerate osseointegration but also enhance the attained level of final-stage osseointegration over time.

Notably, a case series of photofunctionalized implants focusing on complex scenarios reported an unusual but clinically attractive pattern: marginal bone gain (coronal migration of bone contact) rather than loss over 1 year, with mean MBL changing from approximately − 0.35 mm at crown placement to + 0.16 mm at 1 year and no implant showing marginal bone loss during the follow-up interval [144]. While this evidence is limited by small sample size and case-series design, it aligns with the concept that accelerated and strengthened bone–implant integration may favor peri-implant bone maintenance under function [144].

#### Peri-implant soft-tissue parameters, inflammation, and esthetic outcomes

Direct clinical evidence addressing peri-implant soft-tissue health and esthetic outcomes after UV photofunctionalization remains limited compared with the extensive literature on stability and bone-related endpoints. Among the available studies, only a small subset incorporated peri-implant mucosal or esthetic indices as primary or secondary outcomes. In a randomized controlled trial in the anterior maxilla, immediate implants pretreated with photofunctionalization were compared with platelet-rich plasma pretreatment and untreated controls, with follow-up to 12 months and assessment of pink and white esthetic scores (PES/WES) [133]. While photofunctionalization significantly improved implant stability, no statistically significant differences were observed in PES/WES or marginal bone loss between groups. These findings highlight the current limitation of conventional esthetic indices for detecting potentially subtle interface-level biologic differences and underscore the need for more sensitive, barrier-oriented peri-implant soft-tissue endpoints in future clinical studies.

A complementary perspective is provided by a large clinical study that systematically monitored surgical and postoperative complications associated with photofunctionalized implants, including pain, bleeding, inflammatory tissue reactions, delayed or impaired wound healing, infection, and neurosensory disturbances [140]. In this cohort, implant success was defined not only by osseointegration and stability but also by the absence of inflammatory signs and progressive marginal bone loss, thereby explicitly embedding peri-implant soft-tissue health within the clinical success criteria. In the untreated control group, 11 complications were documented: titanium mesh exposure (3 implants), peri-implant infection with soft-tissue dehiscence in a simultaneous guided bone regeneration (GBR) case (1 implant), noninfectious titanium mesh exposure (5 implants), cover-screw exposure (1 implant), and a transient mild sensory disturbance (1 implant). In contrast, the photofunctionalized group showed no surgical complication requiring an additional procedure; only one implant was associated with noninfectious titanium mesh exposure. Overall, the surgical complication rate was 4.95% (11/222 implants) in controls versus 0.59% (1/168 implants) with photofunctionalization [140]. Although quantitative mucosal inflammation indices (e.g., bleeding on probing or mucosal recession) were not systematically reported, the reduced incidence of wound-healing complications and inflammatory adverse events indicates that photofunctionalization does not compromise—and may support—early peri-implant health.

#### Summary of clinical takeaways for peri-implant health

Taken together, the 13 included clinical papers support three clinically relevant inferences:

1. Early osseointegration acceleration is the most consistent clinical signal for UV-photofunctionalized implants, expressed as higher ISQ values and/or faster stability development (OSI) within weeks to months. This advantage is particularly evident in complex indications—such as guided bone regeneration (GBR), sinus elevation, and extraction sockets—where OSI separation between treated and untreated implants is often most pronounced. Similar secondary-stability gains have also been reported in controlled trials both with and without systemic constraints on healing (e.g., diabetes).
2. Peri-implant bone outcomes appear favorable in the early-to-intermediate period, including reduced marginal bone loss in pooled controlled evidence, supportive radiographic patterns in selected complex-case cohorts, and durable long-term success in regular and complex sites with serial radiographic monitoring. In controlled diabetic patients, less distal crestal bone loss at 9 months provides an additional signal consistent with improved bone maintenance.
3. Direct evidence for peri-implant mucosal health is present but comparatively sparse. Only a subset of trials reports peri-implant disease indices (e.g., bleeding on probing, probing depth) and/or esthetic and soft-tissue outcomes, limiting the strength of conclusions about mucosal inflammation control or soft-tissue stability as primary clinical endpoints.

Overall, the clinical literature supports positioning UV photofunctionalization as an interface-first clinical strategy: by accelerating and strengthening early osseointegration, it may shorten the biologically vulnerable period during which micromotion and microgaps can promote marginal bone loss and peri-implant inflammation. At the same time, the field would benefit from more standardized, long-term reporting of mucosal health endpoints and peri-implant disease incidence to fully define its contribution to peri-implant health over time.

## Discussion

### Principal findings: an “interface-first” triad relevant to peri-implant health

**Fig. 8:**
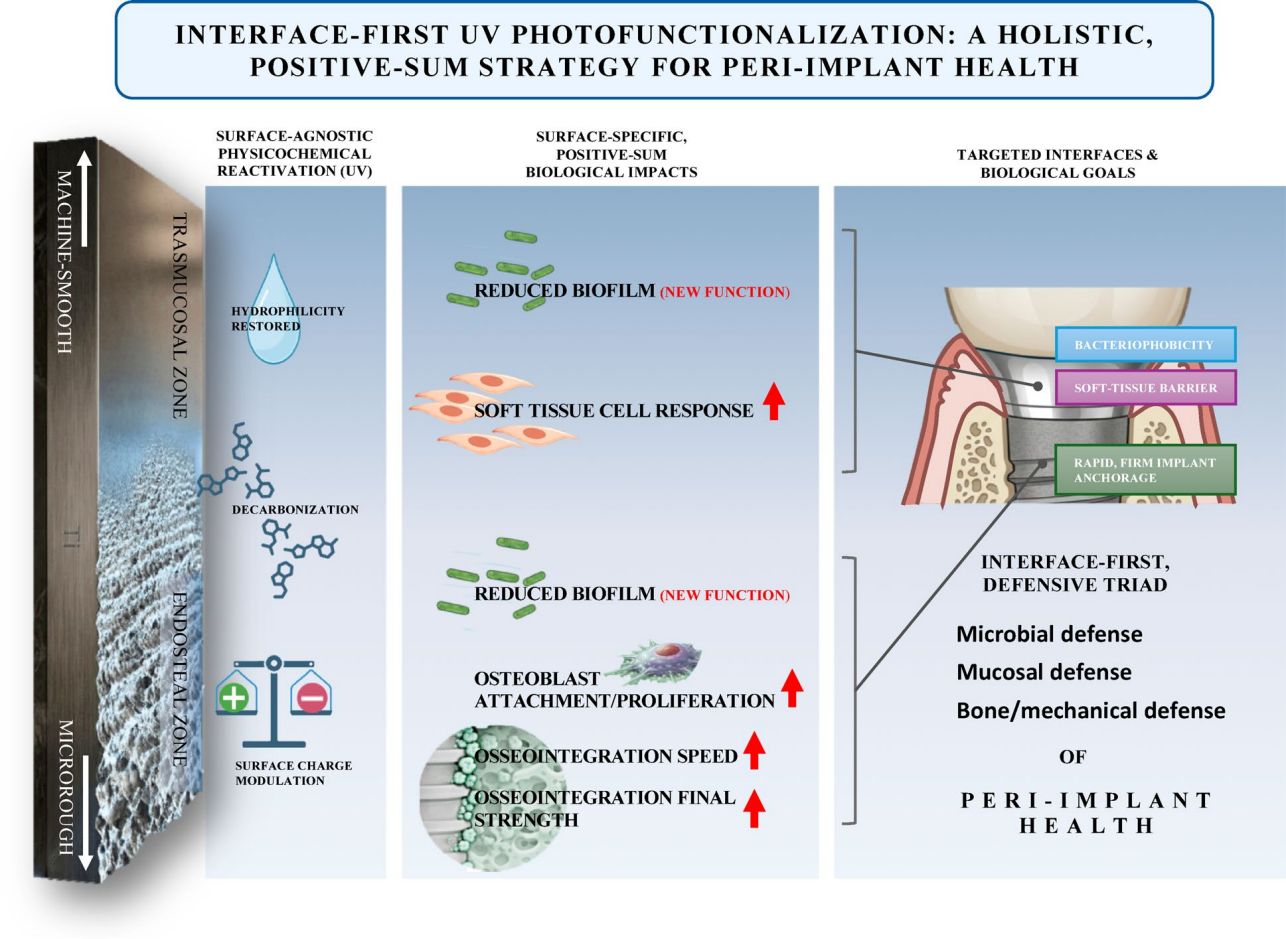
Interface-first, surface-agnostic reactivation by UV photofunctionalization and its zone-specific, positive-sum biological outcomes, enabling the defensive triad of peri-implant health. Summary diagram integrating the key concepts and findings of this review across smooth (transmucosal/abutment) and microrough (endosteal/fixture) titanium surfaces. The left column depicts a surface continuum from smooth (top) to microrough (bottom), emphasizing that UV photofunctionalization is non-destructive and preserves the underlying topography. The second column summarizes the common physicochemical effects shared across surface types, including restoration of superhydrophilicity, hydrocarbon removal (decarbonization), and modulation of the electrostatic surface state. The third column illustrates the surface-specific biological impacts that emerge within each zone. In the smooth transmucosal zone, UV treatment reduces initial bacterial adhesion and early biofilm accumulation (newly acquired bacteriophobicity) while enhancing soft-tissue cell performance, including epithelial integrity and fibroblast attachment/retention. In the microrough endosteal zone, UV photofunctionalization similarly suppresses early biofilm accumulation (newly acquired function) while accelerating osseointegration kinetics (faster stability development) and strengthening interfacial anchorage (stronger bone–implant integration). The right column links these effects to their primary clinical targets: bacteriophobicity and soft-tissue barrier function at the abutment interface, and rapid, firm implant anchorage at the bone–fixture interface. Together, these form the defensive triad of peri-implant health

The clinical relevance of an interface-first framing is underscored by the persistent unmet need in peri-implantitis prevention and management. Across the clinical literature, no single therapeutic approach has emerged as reliably superior across settings and study designs. This reality supports treating anti-biofilm behavior and mucosal barrier biology as co-equal pillars—alongside classic osseointegration endpoints—when synthesizing evidence on implant-surface technologies. From this perspective, the principal findings of the present review are integrated and summarized in Fig. 8.

Across the included evidence base, UV photofunctionalization consistently trends toward three interrelated effects: (i) reduced initial bacterial attachment and early biofilm development, (ii) enhanced soft-tissue cell attachment and retention consistent with improved mucosal barrier formation, and (iii) accelerated stability development of implants. Critically, these three domains converge on a unified translational framework—providing microbial defense, mucosal defense, and bone/mechanical defense, respectively—and together constitute the defensive triad of peri-implant health (Fig. 8).

### Antibiofilm effects: suppressing early bacteria attachment and biofilm initiation

A substantial body of biomaterials literature supports the concept that surface wettability/surface free energy is a primary determinant of early bacterial attachment, sometimes exceeding the contribution of surface roughness. Broad analyses describe a non-linear relationship between surface energy and biofouling (often discussed in the context of the Baier curve), where extremes of wettability—highly hydrophilic or highly hydrophobic—can reduce attachment compared with intermediate wettability states [146]. Recent syntheses similarly conclude that engineered superhydrophilic (and superhydrophobic) states can significantly shift bacterial adhesion and early biofilm development, although the direction and magnitude can vary by organism, conditioning film, and shear environment [147]. This heterogeneity is clinically relevant for peri-implant disease because the oral environment supplies a complex conditioning layer (salivary proteins, blood components during surgery) that can either amplify or dampen a pure wettability effect. Thus, interface physics and chemistry must be considered alongside topography when interpreting antibiofilm outcomes, in which UV photofunctionalization plays a major role.

Within titanium implant literature, the mechanism is often framed as UV-induced conversion from hydrophobic to superhydrophilic surfaces coupled with removal of hydrocarbon contamination, which can reduce “favorable landing zones” for early colonizers [23]. Notably, a systematic review focused on bacterial adhesion to titanium concluded that chemical modifications of surfaces which reduce bacteria-favorable attachment areas can interfere with biofilm formation more strongly than roughness alone, reinforcing a chemistry/energy-first interpretation for early colonization [148].

A particularly important (and sometimes under-emphasized) axis is surface carbon content and hydrocarbon aging. Titanium surfaces naturally accumulate hydrocarbons during storage (“biological aging”). The physicochemical shift is relevant to bacterial adhesion because organic contamination can promote conditioning-film architectures and interfacial energetics that favor microbial retention. In this view, UV photofunctionalization may reduce early biofilm initiation partly by carbon removal itself—a “cleaner” surface that is more uniformly wetted by fluids and less permissive to patchy organic islands that become nucleation points for microcolonies [23].

A broader biomaterials perspective is provided by a recent review of emerging strategies to prevent infection on titanium-based implants, which classifies anti-infective surface approaches into anti-adhesion (bacteria-repulsive) and bactericidal modalities, including superhydrophobic, superhydrophilic, liquid-infused, and polymer-based coatings [149]. Within this framework, UV treatment applied before implantation is positioned as a practical anti-adhesion strategy that can reduce bacterial accumulation without compromising surface biocompatibility. At the same time, this broader literature highlights an important nuance: although wettability is a powerful determinant, surface texture/topography often remains a major modifier of bacterial adhesion. Thus, a “hydrophilicity-only” explanation is likely incomplete, and the observed anti-biofilm phenotype more plausibly arises from coupled effects of surface chemistry/energy, interfacial hydration, and micro-/nano-topography.

### Soft-tissue barrier biology: enhancement of epithelial and fibroblastic attachment, retention, and growth as a peri-implant health lever

A convergent mechanistic theme across soft-tissue–focused photofunctionalization studies is that UV “reprograms” the outermost nanometers of an implant or abutment surface by decomposing and removing surface hydrocarbons. This decarbonization can occur within clinically practical timeframes (e.g., ~ 1 min VUV) and—critically—without introducing new roughness features that could otherwise change soft-tissue behavior or add plaque-retentive morphology [124, 126]. From the broader biomaterials literature, hydrophilic and/or charged surfaces generally support greater mammalian cell attachment and spreading than hydrophobic surfaces in protein-containing environments, consistent with an early “conditioning film → focal adhesion” sequence that is highly sensitive to wettability and surface chemistry [150]. Within photofunctionalization biology, these concepts are often interpreted through the framework of reversing “biological aging,” and this remains mechanistically plausible for soft-tissue endpoints because the earliest steps of fibroblast adhesion depend on adsorption and conformation of extracellular matrix proteins (e.g., fibronectin and collagen motifs) and downstream integrin signaling [151].

At the cellular level, the resulting phenotype is not simply “more cells,” but a more stable and functional adhesion state that resists detachment and supports matrix deposition—features that map directly onto the clinical objective of a durable peri-implant mucosal barrier [124]. Complementary mechanistic readouts implicate canonical focal-adhesion biology, including enhanced integrin–ECM interactions and increased collagen deposition, consistent with more rapid maturation of the connective-tissue attachment apparatus [125]. A particularly compelling mechanistic extension is the proposed formation of a proteoglycan/glycosaminoglycan (GAG)-rich interface after VUV photofunctionalization, supported by (i) enrichment of heparin-/GAG-/sulfur-compound–binding gene clusters and (ii) loss-of-function attenuation of the adhesion advantage using GAG-degrading enzymes [125]. Conceptually, this provides a chemistry-driven route to stronger soft-tissue adhesion at the titanium interface.

Beyond static attachment, UV-treated surfaces also appear to promote dynamic, wound-healing–like behaviors that are relevant to early barrier establishment—both by enabling more effective cell recruitment/migration and by reducing stress and inflammatory tone in fibroblasts encountering the surface. In a transmigration model designed to mimic recruitment across a material boundary, UV-treated titanium increased fibroblast recruitment and transmigration, expanded migration zones, and—most notably—enabled oxidatively damaged human gingival fibroblasts to migrate and spread under conditions where untreated titanium did not. This “functional rescue” aligns with reduced intracellular ROS, preservation of glutathione, and downregulation of pro-inflammatory cytokine expression (e.g., IL1B, IL8) on UV-treated titanium [126]. Importantly, analogous behavior has been reported beyond titanium (e.g., zirconia), suggesting that the core mechanism is not metal-specific but instead linked to carbon pellicle removal and restoration of hydrophilicity across clinically relevant biomaterials [102].

The clinical translation of this chemistry-first effect becomes particularly apparent at the abutment level, where polishing and finishing are routine. Polishing, while intended to reduce roughness-associated plaque retention, is effectively controlled adhesive wear and can chemically contaminate titanium, increasing surface carbon and compromising epithelial attachment. In one study, polished titanium showed reduced epithelial attachment, spreading, and retention, whereas UV treatment removed contaminants (including silicon signals), restored hydrophilicity, and not only reversed the deficit but produced epithelial attachment and retention exceeding that of the original machined surface [129]. This is a key peri-implant health implication: abutment-level bioactivity may be as consequential as fixture-level osteoconductivity, and the mucosal barrier may be most vulnerable precisely where chairside and laboratory manipulation is common—and often unavoidable—creating a practical trade-off between smoothing (for plaque control) and chemical contamination (detrimental for soft-tissue integration). UV photofunctionalization is positioned to negate this trade-off by restoring a bioactive surface state while preserving the benefit of a polished morphology. Consistent with this concept, UV treatment has also been reported to decontaminate titanium impurities introduced even by medical glove contact [152].

### Clinical translation toward peri-implant health: implications, protocol dependence, evidence gaps, and limitations

The clinical literature included in this review supports a consistent signal—accelerated and elevated stability development after UV photofunctionalization—most commonly interpreted as faster and stronger osseointegration. From a peri-implant health standpoint, this signal is clinically meaningful because the early healing window represents a biologically vulnerable period: inadequate primary stability, micromotion, and delayed interfacial bone formation can amplify marginal remodeling and increase inflammatory susceptibility in the peri-implant compartment. In this sense, clinical stability kinetics can be reframed as more than a mechanical metric (see the formation of the defensive triad in Fig. 8). Earlier attainment of a mechanically competent bone–implant interface plausibly shortens the period during which microbial challenge and mechanical perturbation are most likely to initiate a trajectory toward crestal bone instability and peri-implant inflammation. At the synthesis level, a systematic review/meta-analysis of randomized clinical trials likewise concluded that photofunctionalization is an effective and practical strategy to accelerate osseointegration and reduce overall healing time [28].

However, the available clinical datasets still provide an incomplete answer to the central peri-implant health question—whether photofunctionalization supports long-term tissue stability under sustained plaque challenge—because mucosal health outcomes are inconsistently measured and rarely standardized across studies. Most trials and cohorts emphasize ISQ/OSI or radiographic bone levels, with less frequent reporting of bleeding on probing, suppuration, plaque index, mucosal recession, keratinized mucosa width, peri-implant crevicular fluid biomarkers, or microbiome-level changes. Where peri-implant health parameters are reported, they suggest potentially favorable trends in esthetic indices and peri-implant disease outcomes in some controlled datasets, yet heterogeneity in design, follow-up, and outcome definitions remains substantial and long-term disease-incidence endpoints are sparse. Accordingly, the current evidence more strongly supports the statement that photofunctionalization accelerates interfacial stabilization and may strengthen implant–tissue and abutment–tissue defensive interfaces than the stronger claim that it prevents peri-implantitis.

A key reason to expect heterogeneity—and an opportunity for more precise peri-implant health framing—is that photofunctionalization effects are likely protocol dependent. UV band and device spectrum (UVA vs. UVC vs. VUV), exposure duration, and timing relative to placement can plausibly alter the magnitude and persistence of the physicochemical “activation” state [5, 10]. The treated component also matters: treating the fixture may predominantly influence osseointegration kinetics, whereas treating abutment and transmucosal components may more directly affect epithelial and connective tissue attachment, which is central to barrier integrity. Surface type is another likely modifier; in general, photofunctionalization appears to “unlock” the biological potential of the existing surface design rather than substitute for it, and roughened surfaces may show larger osseointegration gains. By contrast, from a soft-tissue perspective, how photofunctionalization interacts with abutment texture/topography remains incompletely defined—reflecting a broader gap in the field, since even without photofunctionalization it is not fully resolved which transmucosal topographies are optimal for durable barrier function (see 2.3. Soft-tissue biology dilemma and Fig. 8). Differences in implant systems, storage/aging conditions before UV treatment, and clinical protocols (immediate vs. early vs. conventional loading; grafted vs. native bone; complex surgical indications) further contribute to variability and should be treated as effect modifiers.

Notably, the biological “quality” of the peri-implant interface—how completely bone occupies the interface, whether a stable marginal “seal” is formed, and how resilient that interface remains under inflammatory challenge—remains largely unmeasured clinically. Two preclinical dog studies provide a useful blueprint for what next-level endpoints could look like. In a dog jawbone model using commercial microrough implants, 15-min photofunctionalization converted the surface from hydrophobic to superhydrophilic and was associated with ~ 50% higher removal torque and significantly higher BIC across marginal, cortical, and marrow zones. Importantly, the authors described an “intensive mineralized layer” localized to the marginal bone exclusively around UV-treated implants, supporting the concept of bone seal with a stronger, more continuous, and more mature interfacial architecture rather than “more bone” alone [153].

The second dog study offers a concrete example of disease-resistance testing that goes beyond standard osseointegration metrics and directly interrogates peri-implantitis vulnerability—an area rarely addressed in clinical photofunctionalization datasets [154]. In a ligature-induced peri-implantitis model, UV-treated implants showed significantly less crestal bone loss radiographically (2.0 ± 0.5 mm vs. 2.7 ± 0.4 mm) and smaller resorption volume by micro-CT (45.7 ± 9.6 mm^3^ vs. 64.4 ± 10.6 mm^3^). Histology suggested maintenance of the bone–implant interface in the UV group, whereas controls exhibited marked cervical resorption and partial interface destruction [154].

Several limitations of the current evidence base should also be acknowledged. Compared with the extensive in vitro and preclinical literature, the available clinical evidence for UV photofunctionalization remains relatively limited in both scale and duration. Existing clinical studies exhibit substantial heterogeneity in implant systems, surface characteristics, UV devices and wavelength bands, irradiation protocols, timing of treatment, loading conditions, and outcome definitions, complicating direct comparison across investigations. In addition, many clinical studies have focused primarily on surrogate endpoints such as implant stability quotient (ISQ), osseointegration speed index (OSI), and marginal bone level changes, whereas long-term peri-implant disease prevention, mucosal inflammatory stability, microbiologic outcomes, and durability of the soft-tissue barrier remain insufficiently characterized. A substantial proportion of the mechanistic and clinical evidence also originates from a limited number of research groups and device/protocol platforms, which may constrain external validity across broader implant systems and clinical environments. Furthermore, several studies involve proprietary implant systems or commercially linked UV platforms, underscoring the importance of independent replication across research groups, implant systems, UV devices, and clinical settings to strengthen translational confidence and establish broader generalizability.

Despite these limitations, the available evidence collectively supports the hypothesis that UV photofunctionalization may enhance not only early integration kinetics but also the structural integrity and defensive capacity of the marginal interface. This concept is directionally consistent with reports of elevated marginal bone levels in clinical case-series observations [144] and with randomized controlled evidence suggesting a more favorable interfacial bone structure after osseointegration [135]. Accordingly, the next generation of clinical research should be designed to test interface quality and robustness—not only early or late stability—using standardized marginal bone change with sufficient follow-up, validated peri-implant inflammatory indices, and biomarker-based endpoints where feasible. It will be especially informative to evaluate photofunctionalization in risk-enriched cohorts (history of periodontitis, smokers, complex grafted sites, reduced primary stability, and systemic conditions associated with delayed healing), so the “stronger and more resistant interface” hypothesis can be examined under clinically relevant challenges. Finally, fixture-only treatment should be directly compared with combined fixture-plus-abutment treatment, because the latter more closely reflects the interface-first logic of peri-implant health (Fig. 8).

### Biological catalyst to provide positive-sum interface

As discussed in Sect.  “Biological trade-offs in implant dentistry” (Biological trade-offs), clinical success in implant dentistry is shaped by a “race to the surface,” in which osteogenic cells, soft-tissue cells, and bacterial colonizers compete to occupy the same interface. Because these actors often favor conflicting surface characteristics—particularly with respect to roughness and plaque retention—implant design has historically required compromises. Achieving a single surface architecture that simultaneously maximizes osseointegration, strengthens the mucosal barrier, and limits early biofilm formation remains a sophisticated and persistent challenge.

In this context, UV photofunctionalization can be viewed as a biological catalyst that partially transcends topographical trade-offs by optimizing surface energy and chemistry in a largely substrate-independent manner. The “substrate” here includes different material classes (commercially pure titanium, titanium alloys, and zirconia) and a wide range of surface textures. Importantly, “surface-agnostic” in this context refers to a topography-preserving physicochemical reactivation principle rather than equivalent levels of biologic or clinical evidence across all materials and outcomes. Current evidence is strongest for titanium-based systems, whereas supportive zirconia evidence is emerging primarily from soft-tissue and interface-focused investigations. Unlike purely topographical strategies that must often choose between “rough” and “smooth” to preferentially benefit one biological outcome over another, photofunctionalization acts as a largely topography-preserving enhancement.

This topography-agnostic mode of action has practical implications across implant zones, as described in Fig. 8. On roughened endosteal surfaces, photofunctionalization can unlock osteogenic potential by converting an aged, hydrocarbon-contaminated, hydrophobic surface state into a high-energy, hydrophilic interface—while also shifting early bacterial attachment dynamics in a direction consistent with reduced initial colonization. On smoother transmucosal or abutment surfaces, where evidence for reduced bacterial attachment is often most pronounced, photofunctionalization likewise enhances bacteriophobic tendencies and improves epithelial and fibroblastic attachment/retention, supporting barrier formation. Although it remains unresolved whether smoother or more textured transmucosal designs are optimal for long-term mucosal stability, photofunctionalization introduces the possibility that future abutment designs could incorporate more deliberate texture to improve soft-tissue retention while maintaining control over early biofilm initiation.

Thus, photofunctionalization helps decouple surface geometry from surface bio-reactivity, enabling a “positive-sum” interface: additive biological function without the structural penalties (Fig. 8). Clinicians and engineers can select the most appropriate topography for each clinical zone (e.g., a smooth transmucosal neck and a rough endosteal body) while using photofunctionalization to maximize the biological affinity of those surfaces and strengthen their defensive profile. Ultimately, defining UV photofunctionalization as a “fresh-start” technology for titanium and zirconia reframes surface optimization as the restoration of a high-energy, decarbonized state—mitigating the adverse effects of biological aging and reducing uncertainty in surface selection. A representative example beyond cell-level biology is the reported marked increase in titanium bonding energy to glass-ionomer cement after UV photofunctionalization [118, 119], underscoring the broader principle that high-energy, decarbonized surfaces can interact more strongly with surrounding biological and material environments.

## Conclusion

UV photofunctionalization is best understood as an interface-first strategy with direct relevance to peri-implant health, rather than an osseointegration-only technology. It represents a surface-agnostic physicochemical reactivation approach—topography-preserving and functionally additive—that restores high surface energy and favorable surface chemistry (via decarbonization and superhydrophilicity) without altering the underlying surface architecture. Importantly, “surface-agnostic” in this context refers to preservation of the original topography and applicability across different surface designs, rather than equivalent levels of evidence across all materials. Current evidence is strongest for titanium, whereas supportive zirconia evidence is emerging primarily from soft-tissue and interface-focused studies. Across the evidence base, UV photofunctionalization consistently demonstrates three interrelated effects: reduced initial bacterial attachment and early biofilm formation, enhanced soft-tissue cell attachment and retention supporting mucosal barrier function, and accelerated and enhanced development of osseointegration. These effects converge into an interface-first defensive triad of peri-implant health—comprising bacteriophobicity (microbial defense), soft-tissue barrier function (mucosal defense), and rapid, firm implant anchorage (bone/mechanical defense). While in vitro and preclinical findings are strongly supportive, current clinical evidence is strongest for accelerated osseointegration and stability development, whereas long-term peri-implant health outcomes—particularly mucosal inflammatory status, microbiologic behavior, and disease-prevention endpoints—remain incompletely defined. Future studies should therefore move beyond stability metrics alone and incorporate standardized peri-implant health–centric outcomes, while also addressing protocol and component dependencies (e.g., fixture vs. abutment, UV spectrum, and timing of application). Collectively, UV photofunctionalization emerges as a practical, zone-adaptable, and holistic interface strategy with the potential to support the defensive triad of peri-implant health, although further longitudinal clinical validation remains necessary to establish its long-term role in peri-implant disease prevention.

## Data Availability

No datasets were generated or analysed during the current study.

## Abbreviations

c.p.: Commercially pure
GAG: Glycosaminoglycan
GBR: Guided bone regeneration
ISQ: Implant stability quotient
OSI: Osseointegration speed index
PRP: Platelet-rich plasma
RFA: Resonance frequency analysis
ROS: Reactive oxygen species
SEM: Scanning electron microscopy
UV: Ultraviolet
VUV: Vacuum ultraviolet
XPS: X-ray photoelectron spectroscopy
